# A Process Mining Pipeline to Characterize COVID-19 Patients' Trajectories and Identify Relevant Temporal Phenotypes From EHR Data

**DOI:** 10.3389/fpubh.2022.815674

**Published:** 2022-05-23

**Authors:** Arianna Dagliati, Roberto Gatta, Alberto Malovini, Valentina Tibollo, Lucia Sacchi, Fidelia Cascini, Luca Chiovato, Riccardo Bellazzi

**Affiliations:** ^1^Department of Electrical, Computer and Biomedical Engineering, University of Pavia, Pavia, Italy; ^2^Dipartimento di Scienze Cliniche e Sperimentali dell'Università degli Studi di Brescia, Brescia, Italy; ^3^Department of Oncology, Lausanne University Hospital, Lausanne, Switzerland; ^4^Istituto di Ricovero e Cura a Carattere Scientifico (IRCCS) Istituti Clinici Scientifici Maugeri, Pavia, Italy; ^5^Dipartimento di Scienze della Vita e Sanità Pubblica, Università Cattolica del Sacro Cuore, Roma, Italy

**Keywords:** healthcare dynamics, digital health, precision medicine, temporal phenotypes, COVID-19, Electronic Health Record (EHR), process mining, electronic phenotyping algorithms

## Abstract

The impact of the COVID-19 pandemic involved the disruption of the processes of care and the need for immediately effective re-organizational procedures. In the context of digital health, it is of paramount importance to determine how a specific patients' population reflects into the healthcare dynamics of the hospital, to investigate how patients' sub-group/strata respond to the different care processes, in order to generate novel hypotheses regarding the most effective healthcare strategies. We present an analysis pipeline based on the heterogeneous collected data aimed at identifying the most frequent healthcare processes patterns, jointly analyzing them with demographic and physiological disease trajectories, and stratify the observed cohort on the basis of the mined patterns. This is a process-oriented pipeline which integrates process mining algorithms, and trajectory mining by topological data analyses and pseudo time approaches. Data was collected for 1,179 COVID-19 positive patients, hospitalized at the Italian Hospital “Istituti Clinici Salvatore Maugeri” in Lombardy, integrating different sources including text admission letters, EHR and hospital infrastructure data. We identified five temporal phenotypes, from laboratory values trajectories, which are characterized by statistically significant different death risk estimates. The process mining algorithms allowed splitting the data in sub-cohorts as function of the pandemic waves and of the temporal trajectories showing statistically significant differences in terms of events characteristics.

## Introduction

The impact of the COVID-19 pandemic on Hospital Care and Health Care Delivery involved the disruption of the processes of care and the need for immediately effective re-organizational procedures (1). Since March 2020, it was clear to the health informatics scientific community the necessity to leverage Electronic Health Record (EHR) data to support critical decisions. In particular, during the pandemic, hospital management and processes had to be transformed at an unprecedented pace and the analysis of EHR may reveal the real impact of such changes (2).

Trajectories mining from EHR data allows to outline emergency guidelines and reveal how single organizations responded to emergency measures, and adopted them on the basis of the treated populations.

An important aspect to be considered are the pandemic waves. As of September 2021 the COVID-19 pandemic has been characterized by several waves. In Italy the first two waves took place in spring 2020 and fall 2020 (3). There is a substantial interest in comparing patients' trajectories hospitalized during the different waves, to understand both the disease course (e.g., changes due to exposure to different treatments, procedures and processes) and changes in healthcare dynamics. In the context of healthcare dynamics and organizational strategies, rigorous process descriptions by means of digital health tools, such as process mining (4) are needed to provide a robust framework for further investigations regarding clinical and physio-pathological aspects of the disease. Thanks to these tools, it is also possible to compare different aspects of the health care processes: changes in the different waves, differences between demographic strata (i.e., sex and age classes) and among patients' phenotypes (i.e., population strata defined by a specific set of diagnoses).

Identifying and comparing patterns in healthcare processes allows characterizing and stratifying an observed cohort, thus defining so-called temporal phenotypes (5). The application of healthcare process modeling has been recognized as an essential step in the analysis of observational health data (6) for multiple purposes. Clinicians could use healthcare processes models during their practice as part of the move toward precision medicine by identifying subpopulations that have distinct healthcare process patterns after a new diagnosis or change in treatment strategy (7–9). Within clinical research the combination of patient pathophysiology and healthcare processes create a much clearer picture of the overall health status of a patient than either one alone. Furthermore - and in the light of identifying successful approaches for the management and containment of the pandemic and its socio-economic impacts - policy makers can study healthcare processes to identify disparities in access to healthcare among specific populations or to track if regulatory changes are having their expected effects on healthcare processes [https://euprevent.eu/periscope/].

This paper is aimed at providing mining tools that can be applied in the context of digital health to determine how a specific patients' population reflects into the healthcare dynamics of the hospital, to investigate how patients' sub-group/strata respond to the different care processes and to generate novel hypotheses regarding the most effective healthcare strategies.

Nowadays, available process mining tools are mainly focused on qualitative processes descriptions rather than on quantitative processes comparison, suggesting the need to integrate inferential statistical approaches (10). Furthermore, when applying process mining in the health care context to analyze its dynamics, we often face several issues related to the context complexity and events variability. Novel process mining approaches provide solutions to those issues, (11) and should be used in synergy with other methodological approaches (e.g., canonical statistical analysis).

In this work, we propose an analysis pipeline based on time series and process-oriented analysis, which integrates visual analytics, inferential statistics with process mining approaches. The pipeline is aimed at stratifying a population of interest on the basis of several exposure factors, including both clinical processes and patient characteristics. The methods included in the proposed version of the pipeline can be easily applied to real clinical scenarios and provide explainable solutions useful for clinical practice to reply to urgent clinical questions.

The analyses described in this paper have been performed on data from COVID-19 patients hospitalized at Istituti Clinici Scientifici Maugeri (ICSM) in Lombardy region, Italy.

ICSM is a network of hospitals, focused on patients' rehabilitation, therefore no emergency units or intensive care units (ICU) are present. Since the beginning of the outbreak, ICSM repurposed internal wards to treat COVID-19 patients during the acute phase of the disease but not needing intensive care or patients recovering after the acute phase of the disease. Starting from these observations, ICSM patients are peculiar when compared with the COVID-19 patients' population for at least two reasons. First of all, post-acute patients' affluence was mainly driven by the nearby acute hospitals that were not able to face the ever-increasing demand of hospitalizations. Secondly, the lack of ICU excluded hospitalizations of patients with extremely severe clinical manifestations during the acute phase of the disease. On the other hand, the rehabilitative connotation of ICSM offers the opportunity to follow up patients' condition for longer periods compared to acute hospitals.

## Materials and Methods

### Study Population

Our analyses were performed on data collected from 3 hospitals belonging to the ICSM network in the Lombardy Region: Pavia, Lumezzane (in the Brescia area) and Milano. The study included hospitalized patients who had a SARS-CoV-2 Reverse Transcription Polymerase Chain Reaction (RT-PCR) positive test result recorded from February 2020 until August 2021. For patients with multiple RT-PCR tests, only the first positive test was used. Data starting from 2 years before the first positive SARS-CoV-2 test to the last available follow up were considered for each patient. Data were available for 1,179 hospitalized patients with SARS-CoV-2.

### Data Acquisition and Preprocessing

**Figure 1:**
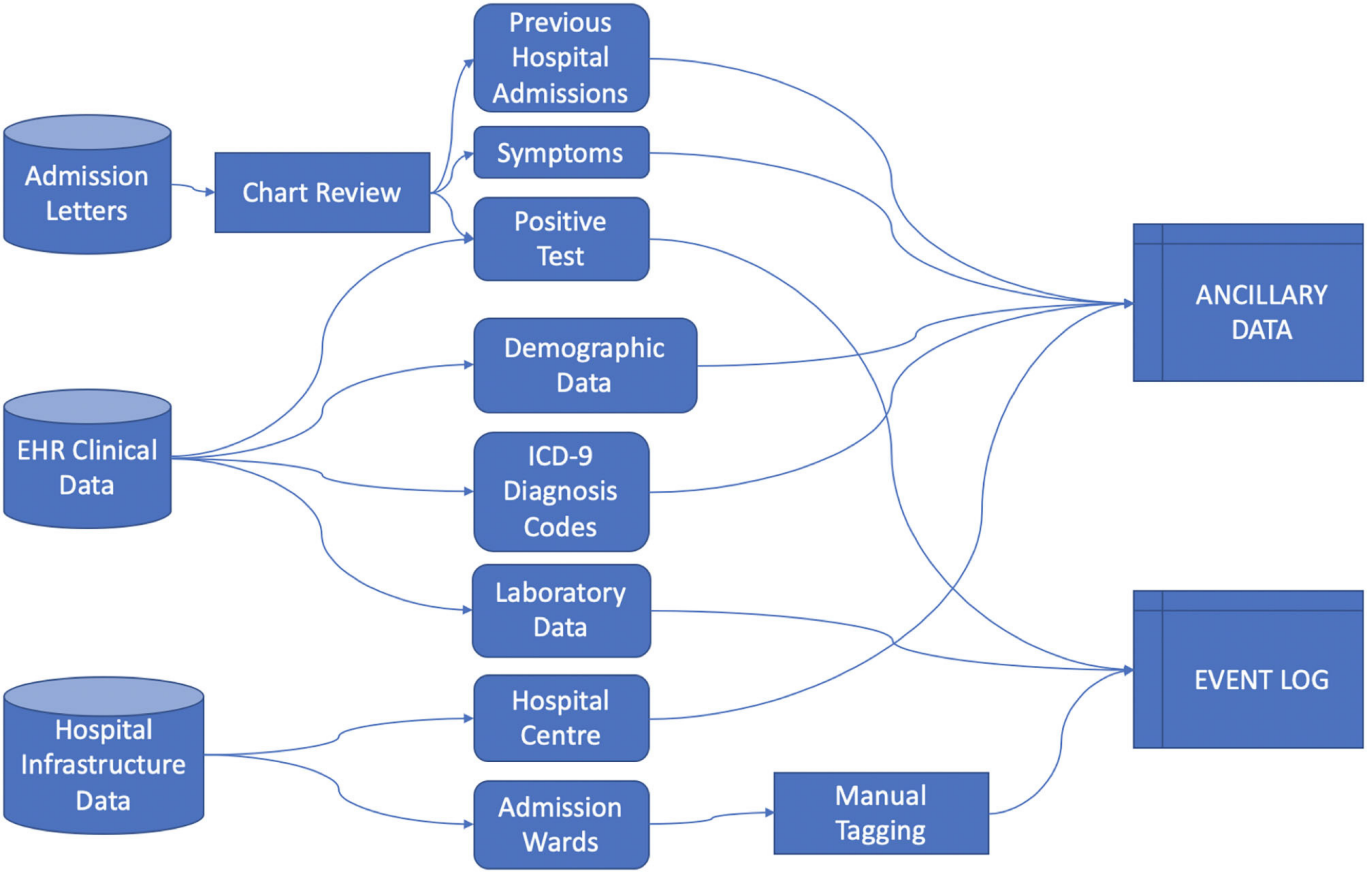
Data acquisition schema.

Data was collected from the ICSM Hospital Information System (HIS) integrating different sources including Text Admission Letters, EHR (including demographic and clinical data) and Hospital Infrastructure Data, from which we extracted events information regarding the hospitals centers and their care processes organization, as illustrated in Figure 1.

We rely on the 4CE consortium data model (12) to collect Demographic data, Diagnosis ICD-9 codes and Laboratory values. Each data source required extensive pre-processing, especially in manually retrieving data from admission letters via chart review, aggregating Diagnosis ICD-9 codes, mapping Laboratory tests to LOINC codes and defining the process events granularity. We defined the index date as the first hospital admission - including a PCR positive test - date and defined “days since admission” as the number of days since the index date.

Healthcare Process data include the events a patient undergoes during their medical history. In this study we included events with a coarse granularity, that is, inpatient stays identified by the ward where the patient was hospitalized. Events were collected from the first positive SARS-CoV-2 test to the last discharge, as recorded by August 2021. Process data were collected in the event log file (13), where each row is referred to a single event and each column includes the patient's ID, time interval of occurrence of the event and the event label.

Ancillary data refer to metadata associated to the event (i.e., the hospital center where the hospitalization occurred, the calendar time of the event associated to the pandemic waves) and to the patient (i.e., age at admission, sex, diagnosis and laboratory test results). Ancillary data can be cross-sectional or longitudinal. In the latter case they are aligned to the event log using the “days since admission” reference.

Along with data cleaning and quality checks, data processing includes the definition of the pandemic waves, disease progression stages and patients' frailty.

We defined the pandemic waves according to the global and local pandemic evolution and consequent ICSM hospital reorganization. We partitioned patients into first and second-wave cohorts according to their first admission to the hospital and relied on the approach exploited in (14) where, although different regions had slightly varying temporal trajectories in COVID-19–related hospitalizations, data indicated 2 predominant waves of hospitalizations, which were used to partition patients as follows: a first wave from January to June 2020, and a second wave from July 2020. We observed a substantial consistency among global and local patterns, where the last admission in COVID wards for the first wave was observed in 2020-06-10 and the first one for the second wave was in 2020-10-02. Thus, we leverage on quarters granularity: the first wave includes 2020 first two quarters (until and including 2020-06-30), the second wave includes 2020 third and fourth quarters and 2021 first three quarters (after 2020-06-30). Note that we merged the 2nd and 3rd wave as novel healthcare processes were established, and maintained, after the first wave.

Disease progression stages were defined in temporal windows computed on the basis of the index date and “days since admission” reference: pre-acute stage (before−7 days), acute stage (from−6 to 30 days), post-acute stage (from 31 to 90 days) and long stage (after 91 days). The weighted Charlson Comorbidity Index (15) was computed via the R package “comorbidity” (16) to measure comorbid disease status during the disease progression.

To define comorbidity phenotypes, we mapped the ICD-9 diagnosis codes to a unique phenotype code (PheCode) (17) and analyzed higher-level categories including: endocrine/metabolic, mental disorders, circulatory system, injuries & poisonings, neurological, neoplasms, genitourinary, musculoskeletal, hematopoietic, digestive and dermatologic diagnosis. While focusing on comorbidities, we excluded diagnoses identifying COVID-19 phenotypes: U07.2 ICD-9 codes and codes mapped to respiratory (Pneumonia and Respiratory failure, insufficiency, arrest) and infectious diseases (Viral Infection), which were recorded across the entire cohort (see the Supplementary Table 2).

A total number of 16 laboratory tests: Alanine Aminotransferase, Aspartate Aminotransferase, Albumin, Cardiac Troponin, C-Reactive Protein (CRP), Creatinine, D-dimer, Ferritin, Fibrinogen, Lactate Dehydrogenase (LDH), Lymphocyte, Neutrophil, Procalcitonin, Prothrombin Time (PT), Bilirubin, white blood cells (WBC), previously included in (12) were analyzed. A preliminary pre-processing phase involved mapping internal coding to the corresponding LOINC identifier (including measurement unit conversion if needed), missing values removal, averaging of multiple measures by day for each patient.

### Process-Oriented Analysis Pipeline

We developed an analysis pipeline based on the heterogeneous collected data (event logs and cross-sectional and longitudinal ancillary data) aimed at identifying the most frequent healthcare processes pattern, jointly analyzing them with demographic and physiological disease trajectories, and stratify the observed cohort on the basis of the mined patterns. This is a process-oriented pipeline which integrates process mining algorithms, time series analysis and visual analytics.

#### Descriptive Analysis

Event and ancillary data were exploited to describe the population characteristics and its evolution during the calendar time and waves. Processes and Clinical characteristics were compared among waves. Each subject was assigned to only one wave considering their first test. Pearson Chi-square (χ2) test for independence was applied to test the null hypothesis of independence between categorical variables, being the expected frequencies always higher than 5 and the remaining assumptions underlying the Pearson Chi-square test met in all cases (18). Given their non-normal distributions (see Supplementary) continuous variables distributions (i.e., Age and Charlson Score) were compared among subgroups by the non-parametric Mann–Whitney test. Categorical variable distributions are described as absolute and relative (%) frequencies. Continuous variables are described by both Mean (standard deviation, SD) and Median (Interquartile Range, IQR: 25–75 percentiles).

Population characteristics were analyzed across calendar time. For each year quarter, starting from the first quartile of 2020 (January, February and March), we computed the proportion of the sex, age quantiles, and center of the first admission strata. Cox proportional hazard regression and kernel density estimation was exploited to study patients' survival and compare it between waves.

Cohort frailty, comorbidity and phenotypes were studied during the different disease stages (pre, acute and post-acute) and stratified by calendar time. Charlson score at each disease stage was compared among the cohorts hospitalized in year quarters. The prevalence of PheCode categories were computed in disease stages and stratified by age classes. PheCode prevalence rate was calculated as the number of persons having a specific diagnosis code registered for the first-time during a given time period (i.e., disease stage time window) divided by the population size during the same time period.

Events distributions were compared by age classes, sex, waves and admission centers.

We exploited Kaplan-Meier curves to describe how long a cohort of patients was in charge of a specific hospital ward or to represent the time needed to move from a state to another (e.g.,: from ‘positive covid test' to ‘admission in a covid unit'). Giving the appropriate meaning to the event and to censor, this tool allows to easily verify, with a logrank test, if sub cohorts (e.g.,: defined by sex, age, etc…) are characterized by statistically different behaviors. Assumptions (19) have been verified (i.e. censoring was unrelated to prognosis, survival probabilities were the same across Waves - see Supplementary Table 7 - and the events happened at the times specified).

The significance level has been set to α = 0.05. Descriptive analyses were performed by R software environment for statistical computing and graphics version 4.1.2 (www.r-project.org).

#### Physio-Pathological Trajectory Mining

To depict the underlying physio-pathological progression of the diseases we mined patients' trajectories from laboratory values, using an approach based on the topological representation of data and pseudo time, as described in detail in (20). These unsupervised temporal models capture continuous changes in disease over time. Identified key temporal features allow characterizing disease subtypes that underpin these trajectories, thus allowing cohort stratification.

We applied the previously developed algorithm to identify the most relevant trajectories. Starting from a patient similarity matrix, computed via cosine similarity, the Topological Data Analysis (TDA) algorithm infers temporal phenotypes from topological models that learn disease states from multiple laboratory measures, then pseudo time series approaches that identify transitions among these states are applied.

Each subject is assigned to a unique trajectory, thus we compared categorical characteristics (sex, wave, admission center) by Pearson chi square test for independence and numeric continuous ones (age and Charlson Score) by Kruskal-Wallis test.

We investigated whether the mined patients' trajectories were predictors of survival. To this end, we have carried out a multivariate survival analysis by using Cox proportional hazard regression to predict in-hospital death during the observation period. Verification of the Cox Regression model assumptions is reported in the Supplementary.

#### Events Process Mining

Process Discovery was performed exploiting the Careflow Miner (CMF) algorithm, as implemented in pMineR (4). Such implementation enriches the original version of the algorithm with a set of features aimed at joining the benefits of Process Discovery with the benefits of the inferential statistics. To do that a CMF graph can be stratified on the base of an attribute (one of the additional columns in the Event Log): this causes the creation of two CFMs which can be compared node by node testing if the number of patients passing through that node are statistically different (with a Fisher's exact test or a Pearson Chi-square test, depending on the observed cardinality). Tests assumptions were verified as each subject can follow one and only one of the compared processes. Similarly, the time needed to reach each node can also be compared, by testing via Mann-Whitney test if the distribution of the times related to two cohorts differs. Additional analyses may include as an example the comparison of probability to reach a final state (such as Death) between cohorts. Sample sizes depend on the applied CFM thresholds, in this application we choose a 12 patients' threshold (i.e., each node accounts for 12 or more observations)

In our analyses we compared CFMs between waves, to study changes in process during the pandemic, and among temporal phenotypes, as mined from trajectories (see 2.3.2), to understand possible correlations between process of care and disease progression.

Analyses were performed by the R statistical tool, version 4.0.2. Data are presented as the main effect estimate with 95% confidence intervals (95% CIs), and the 5% significance level was used for main inferences.

## Results

### Cohort Characteristics and Descriptive Statistics

Data were available for 1,179 inpatients with COVID-19. Table 1 reports demographic characteristics (age at the first positive PCR test), survival at August 2021, first admission hospital center and Charlson Comorbidity Index, stratified by pandemic wave. Patients were assigned by a wave according to their first hospital admission. When comparing First to Second wave, high significant degree of evidences were found in terms of sex (*p* = 0.023, χ2 = 6.1241), age at first test (*p* = 1.05e-08, eff.size = 0.167), center of admission (*p* = 8.964e-05, χ2 = 18.64) and Charlson Comorbidity Index at admission (*p* = 1.52e-20, eff.size = 0.27) distributions.

**Table 1:** Population distribution in waves.

	**First Wave (*N* = 651)**	**Second Wave** **(*N* = 528)**	***p*-value**
Sex			0.023
Female	275 (42.2%)	259 (49.1%)	
Male	376 (57.8%)	269 (50.9%)	
Age			1.053e-08
Mean (SD)	71.9 (13.5)	76.0 (12.8)	
Median [IRQ]	74.0 [63.0, 82.0]	80.0 [70.0, 85.0]	
Survival			0.928
Survived	578 (88.8%)	467 (88.4%)	
Death	73 (11.2%)	61 (11.6%)	
Admission Center			8.964e-05
Lumezzane	182 (28.0%)	140 (26.5%)	
Milano Camaldoli	170 (26.1%)	197 (37.3%)	
Pavia	299 (45.9%)	191 (36.2%)	
Charlson Score at Admission			< 2.2e-16
Mean (SD)	1.16 (1.77)	2.85 (3.01)	
Median [IRQ]	1.00 [0, 2.0]	1.00 [0, 6.0]	

*Data are presented as absolute and relative (%) frequencies or mean (Standard Deviation, SD) and median (25th - 75th percentiles, Interquartile Range, IQR) by wave as appropriate*.

#### Ancillary Data

Quarters are reported in the following as “year.quarter”. The number of subjects hospitalized in quarters are 248 in the first quarter of 2020 (2020.1), 381 in 2020.2, 44 in 2020.3, 388 in 2020.4 and 219 in 2021.1. Note that the total number exceeds 1,179, as one patient can be hospitalized for more than one quarter (see Supplementary Table 1).

**Figure 2:**
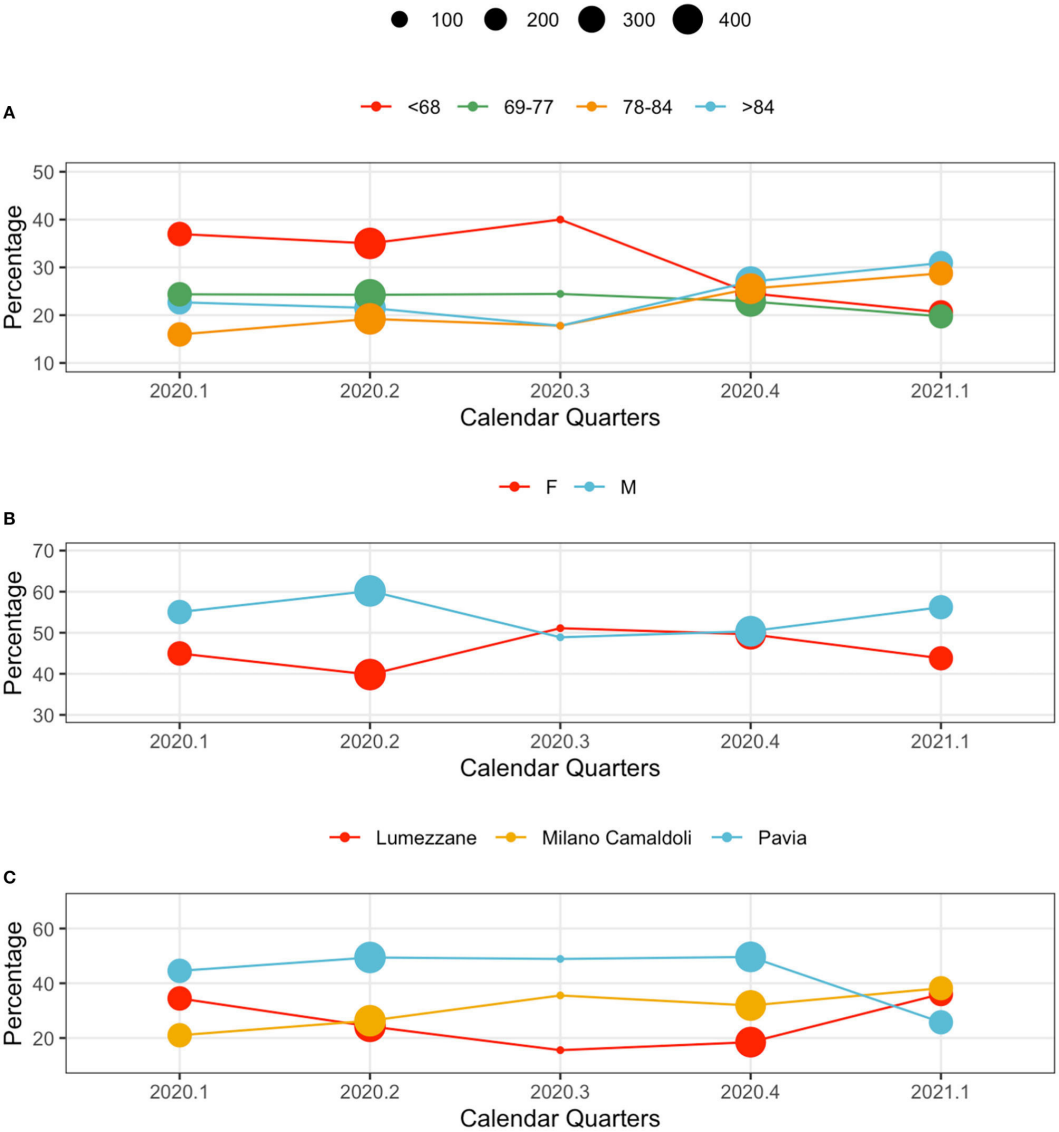
Population characteristics in calendar quarters. From the top: **(A)** age classes, **(B)** sex, and **(C)** centers. The dot size indicates the total number of patients hospitalized in the quarter. The y axis reports the relative frequency (%) of each variable's values by calendar quarter.

Hospitalized population characteristics, along with calendar quarters, are reported in Figure 2. The dots position indicates the percentage of the age, sex and center classes over the total size of population hospitalized in the year quarter. The dot size indicates the total number of patients hospitalized in the quarter. Age classes are computed on the basis of the Age at the first positive test quantiles.

Younger subjects were more frequently hospitalized in the first wave: patients younger than 68 years represented the majority of observations in 2020.1, 2020.2 and 2020.3. This trend changed during the second wave, when elderly subjects (over 84 year) were the majority in 2020.4, and 2021.1 (Figure 2A).

The sex distribution characterizing hospitalized patients show consistently higher percentages of male patients. A slight change occurred during 2020.4, when the demographic of the population was more homogeneous (Figure 2B).

Hospital loads depend on each center's capacity: Pavia (426 beds), Lumezzane (149 beds), Milano Camaldoli (200 beds). Of note, only a portion of each hospital capacity was dedicated to COVID-19 patients based on the geographic zone and on the pandemic-related hospitalizations pressure that varied from week to week. However, patient distributions in centers change during waves, possibly indicating changes in policies and cohort management. During the late second wave (2021.1) patients were evenly distributed across centers (Figure 2C): this aspect is particularly interesting given the rehabilitation nature of the ICSM hospitals. Counts and percentages are provided in Supplementary Table 3.

**Figure 3:**
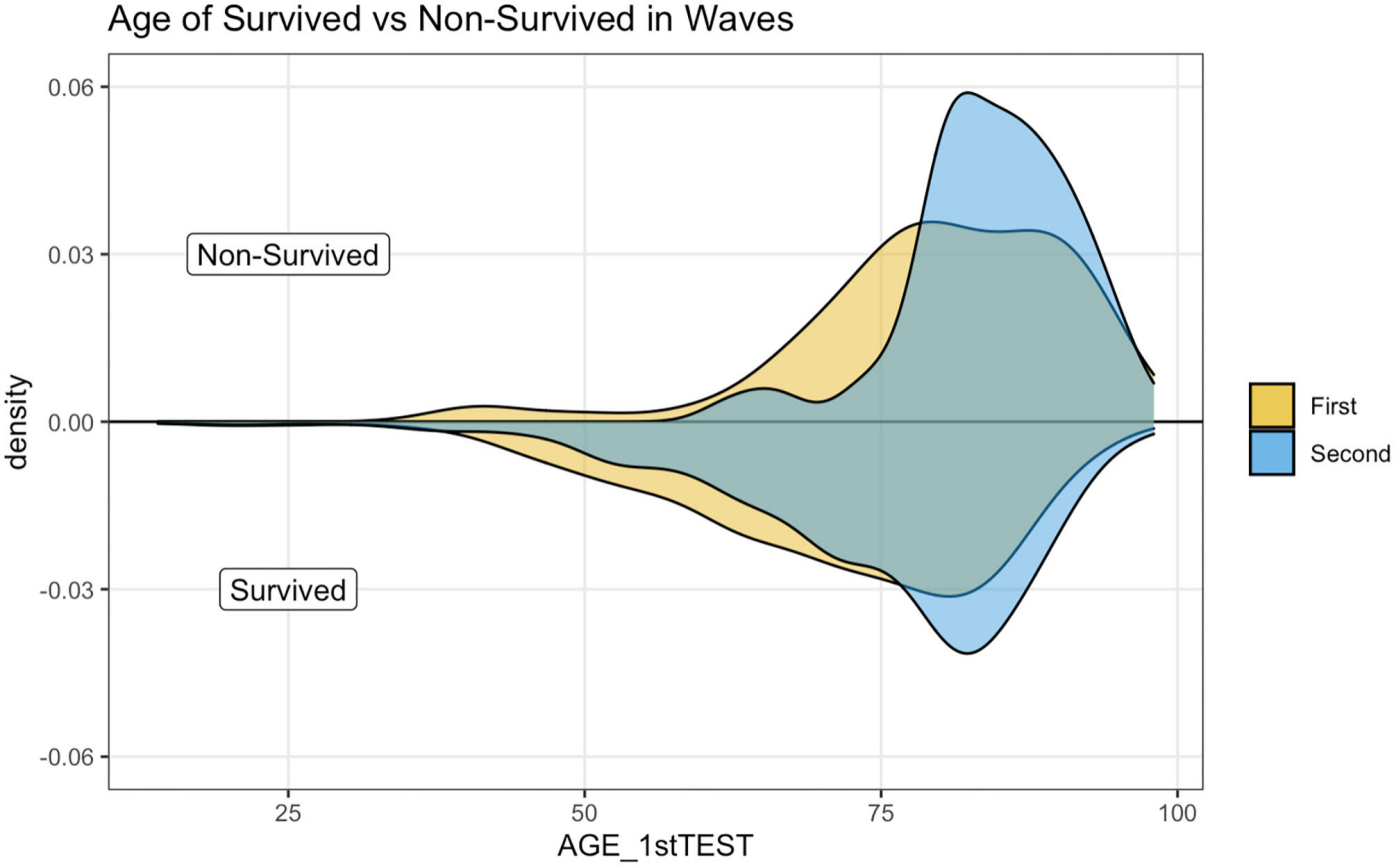
Age distribution by wave in survived and non-survived patients.

Figure 3 illustrates a kernel density estimation that allows comparing age distribution of survived and not survived patients between waves. The top panel indicates how non-survived seems younger during the first wave.

**Figure 4:**
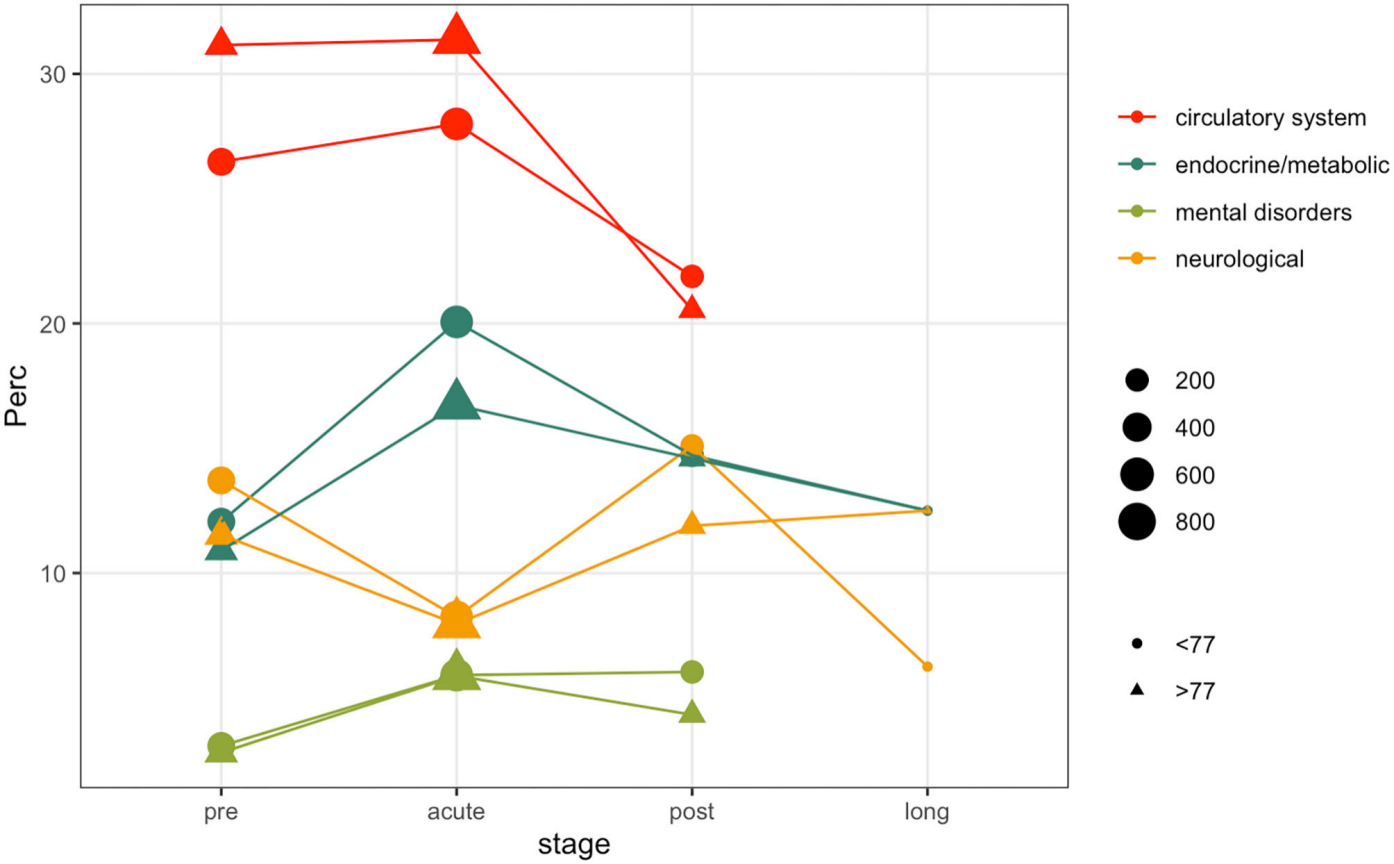
Comorbidity phenotypes prevalence in disease stages stratified by age.

Patients comorbidities and frailty status were investigated leveraging ICD9-CM diagnosis codes collected during each hospitalization. Figure 4 illustrates the prevalence rate of most common non-respiratory PheCodes in specified disease stages. The size of the filled symbols indicates the total number of subjects observed in each stage, the position on the vertical axis indicates the prevalence of the phenotype in the time windows, stratified by age class. For simplicity, we only used two age classes, computed on the median of the age at the first positive test quantiles (i.e., 77 years). Circulatory system phenotypes account for the majority of the comorbidities phenotypes during all the disease stages, being more frequent in older subjects. As in other Phecodes, it is possible to observe a loss-of follow ups in the post and long stages. Endocrine and metabolic phenotypes have an increasing trend in both the age classes during the acute phase, while the trend is decreasing for genitourinary diagnosis. Neurological phenotypes follow another trend, decreasing during the acute stage and increasing in the post stage. Complete counts are provided in Supplementary Table 5.

#### Event Data

**Figure 5:**
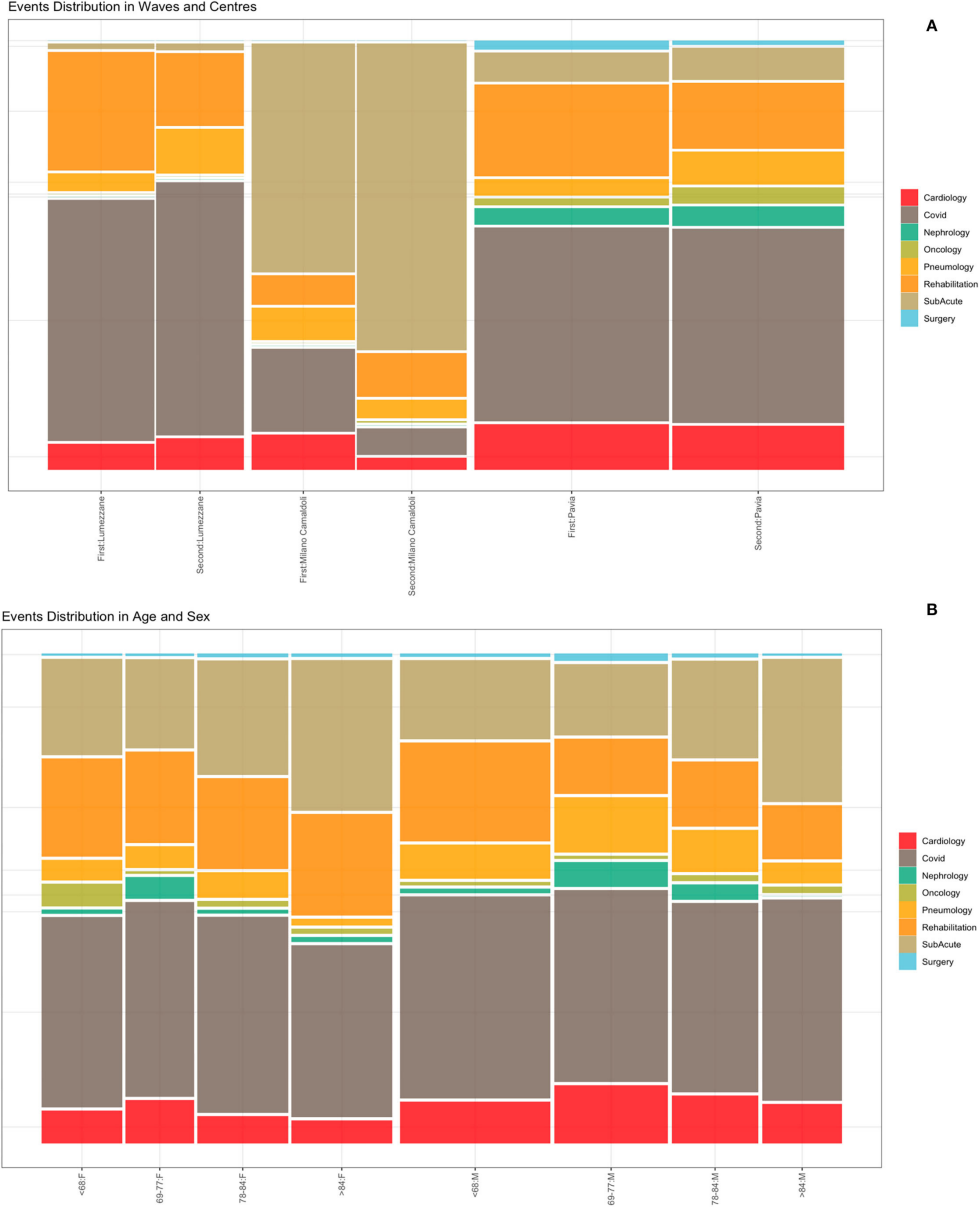
Events distribution by waves and centers **(A)** and by age and sex classes **(B)**. Events consist of hospitalizations.

Events were extracted from the ICSM HIS and depicts the patients careflow in terms of hospitalization wards. The analyzed event log includes a total of 5,176 events (i.e., hospitalizations), whose distributions in waves and population strata are shown in Figure 5. Figure 5 does not include events “Tested Positive” and “Discharge,” recorded for all the 1,179 subjects. Events were collected from February 2020 to August 2021. Supplementary Table 6 reports events counts.

Figure 5A illustrates the distribution of events by center during the two waves. Given the differences in centers organization and pre-pandemic hospitals facilities (i.e., sub-acute, surgery or rehabilitation units), it is possible to observe that the “Milano Camaldoli” center accounts for the majority of sub-acute hospitalization, while the “Pavia” center has more heterogeneous events, including hospitalizations in Surgery, Nephrology and Oncology wards. Figure 5B shows events distribution by sex and age classes. Older subjects (over 84 years) were more frequently hospitalized in the sub-acute units. The age class from 69 to 77 years include subjects that were more frequently admitted in non-covid units, such as cardiology and nephrology wards.

**Figure 6:**
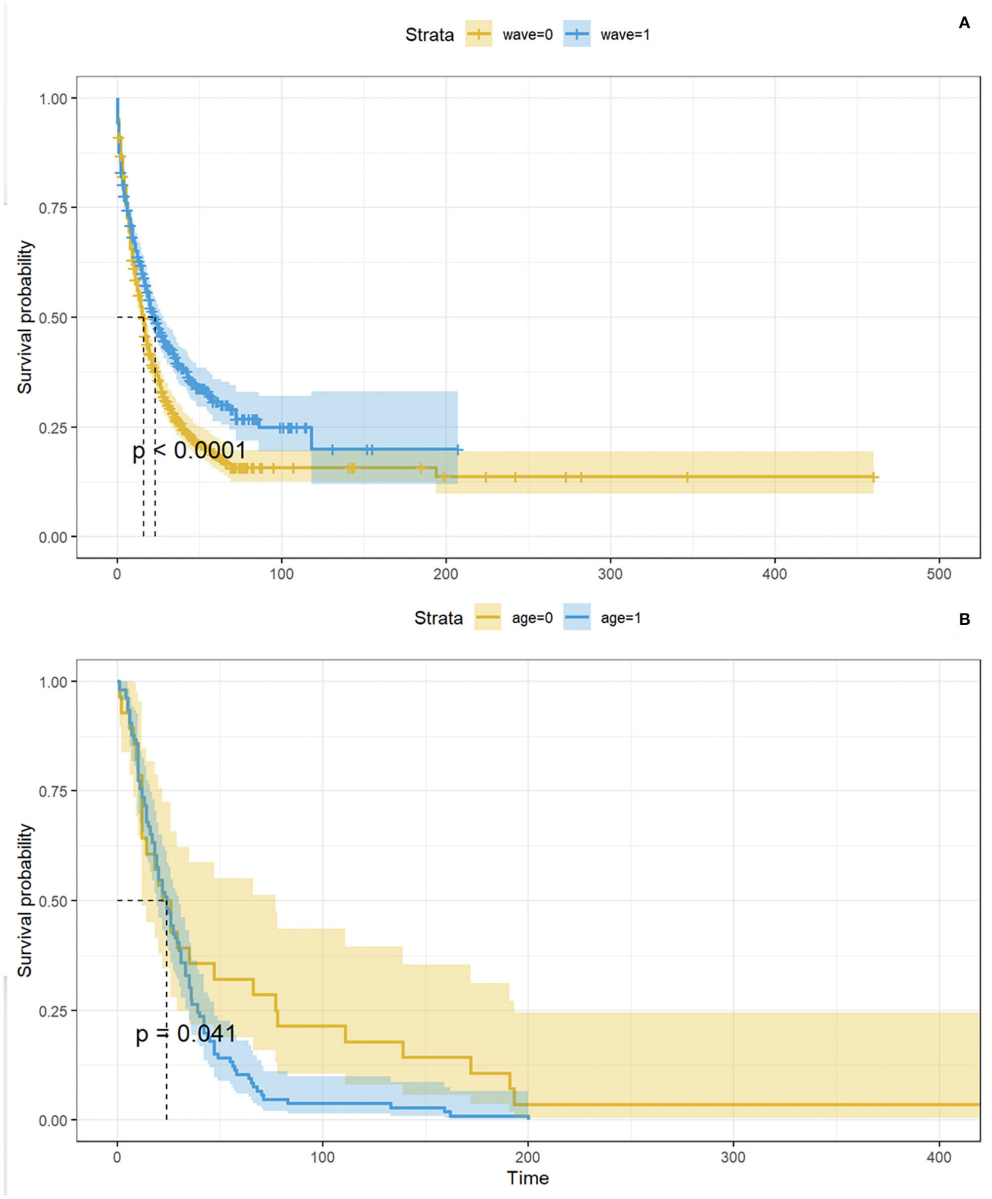
In **(A)**, KM curves showing time between positive test and admission to the ICSM COVID-19 ward by wave. The *p*- value derives from the logrank test. In **(B)**, KM curves showing time between positive test and admission to the ICSM COVID-19 ward by age (<77 and ≥77 years). The p - value derives from the logrank test.

The Kaplan-Meier (KM) curve in Figure 6A compares the time between a patient's COVID-19 positive test and the admission to the ICSM COVID-19 ward. Not all the patients were admitted in the ICSM COVID-19ward because in some cases (e.g., a patient that was hospitalized in a different hospital during the acute phase) the patient was treated in a different ICSM ward for rehabilitation. The logrank test confirmed a statistically significant difference in terms of time to admission to the ICSM COVID-19 ward between the two waves (*p* < 0.0001), with patients from the second wave showing longer time to transfer. Figure 6B shows the time from COVID-19 positive test to death based on data from deceased patients as function of age: older patients showed significantly shorter survival time compared to young patients (*p* < 0.05).

### Patients Trajectories

**Figure 7:**
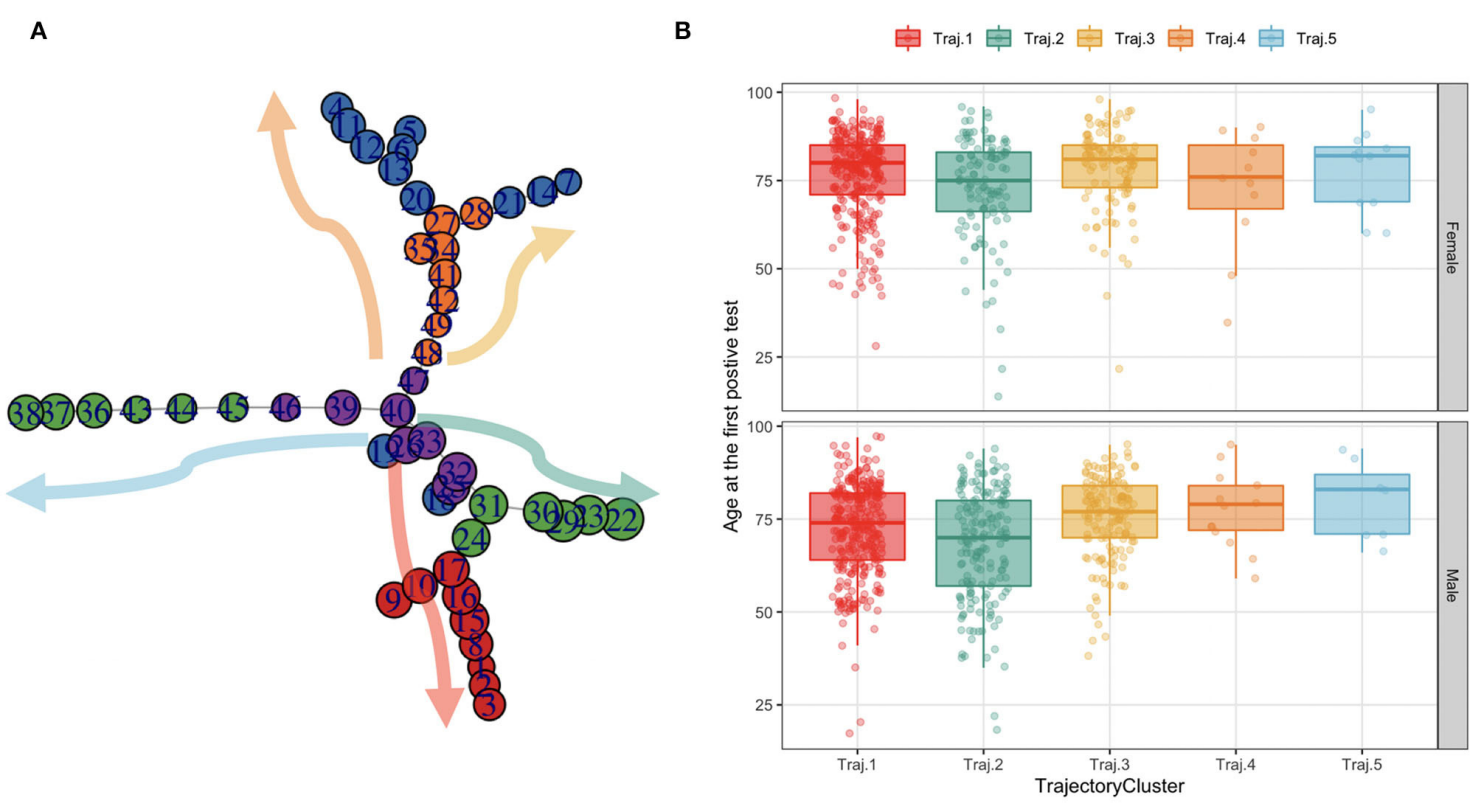
The trajectories mined from the topology and their age distribution in females and males. **(A)** Each node in the graph on the leftmost side represents a cluster of data points as observations in time (i.e., a measure of a lab value). The node coloring is based upon topological structure membership. **(B)** The boxplots on the rightmost side represent the age at the first SARS-CoV-2 PCR test distribution by trajectory in females and males.

The TDA algorithm identified five potential trajectories, from the laboratory values registered during patients' acute hospitalizations and follow-ups. Figure 7 reports the mined trajectories. Each node represents a cluster of data points as observations in time (i.e., a measure of a laboratory value). The node coloring is based upon topological structure membership. The algorithm identifies five distinct trajectories; all of which start from a central cluster which accounts for the first observations in time and progressing toward other clusters in the tips of the topology, as indicated by the arrows. For example, for a subject belonging to the yellow trajectory (Trajectory 3), the disease progression models indicate a transaction from the central nodes to the ones on the top right of the figure. The cohort was then stratified on the basis of the trajectory each patient was assigned to, consisting of five temporal phenotypes that show the progression of each trajectory (each one representing a temporal phenotype) toward the disease's deterioration and distinct outcomes.

We characterize these phenotypes using relevant clinical features values at baseline. Table 2 reports demographic characteristics, survival at August 2021, first admission hospital center and Charlson Comorbidity Index, stratified by trajectory clusters. Trajectories 4 and 5 include a small number of patients (*n* = 24 and *n* = 19 respectively), thus they will be described but not included in the process models' analyses described in Mined Careflows and Comparison in Waves and Temporal Phenotypes. Among the larger groups (trajectories 1, 2 and 3), trajectory 3 includes the older patients, with higher frailty scores at the first admission and percentage of death events in the observation period, opposite to trajectory 2, which includes younger subjects and lower percentage of deceased patients. While accounting only for 19 patients, Trajectory 5 includes the oldest subset of patients.

**Table 2:** Population distribution in trajectories clusters.

	**Traj.1**	**Traj.2**	**Traj.3**	**Traj.4**	**Traj.5**	***p*-value**
	**(*N* = 583)**	**(*N* = 286)**	**(*N* = 254)**	**(*N* = 24)**	**(*N* = 19)**	
Sex						0.058
Female	283 (48.5%)	114 (39.9%)	108 (42.5%)	11 (45.8%)	12 (63.2%)	
Male	300 (51.5%)	172 (60.1%)	146 (57.5%)	13 (54.2%)	7 (36.8%)	
Age						2.454e-06
Mean (SD)	74.4 (12.4)	70.1 (15.0)	76.6 (11.6)	75.0 (14.0)	78.8 (10.7)	
Median [IRQ]	77.0 [67.0, 83.0]	72.0 [61.0, 82.0]	79.0 [71.0, 85.0]	77.5 [70.5,84.5]	82.0 [70.0, 85.0]	
Survival						< 2.2e-16
Survived	555 (95.2%)	273 (95.5%)	178 (70.1%)	12 (50.0%)	17 (89.5%)	
Death	28 (4.8%)	13 (4.5%)	76 (29.9%)	12 (50.0%)	2 (10.5%)	
Charlson Score at Admission						0.00748
Mean (SD)	1.69 (2.30)	1.99 (2.69)	2.31 (2.80)	3.04 (3.63)	1.63 (2.06)	
Median [IRQ]	1.00 [0, 2.00]	1.00 [0, 3.0]	1.00 [0, 3.0]	1.50 [0, 6.0]	1.00 [0, 2.00]	
Wave of the first Admission						0.306
First	332 (56.9%)	146 (51.0%)	146 (57.5%)	12 (50.0%)	8 (42.1%)	
Second	251 (43.1%)	140 (49.0%)	108 (42.5%)	12 (50.0%)	11 (57.9%)	

*Data are presented as absolute and relative (%) frequencies or mean (Standard Deviation, SD) and median (25th - 75th percentiles, Interquartile Range, IQR) by wave as appropriate*.

When comparing trajectories, medium degree of evidences were found for sex (*p* = 0.058, χ2 = 9.1293, df = 4), high degree of evidences were found for age at first test (*p* < 2.2e-16, eff.size = 0.023), deaths (*p* < 2.2e-16, χ2 = 162.13, df = 4), Charlson Score at admission (*p* = 0.00748, eff.size = 0.008), none in in waves (*p* = 0.306).

**Figure 8:**
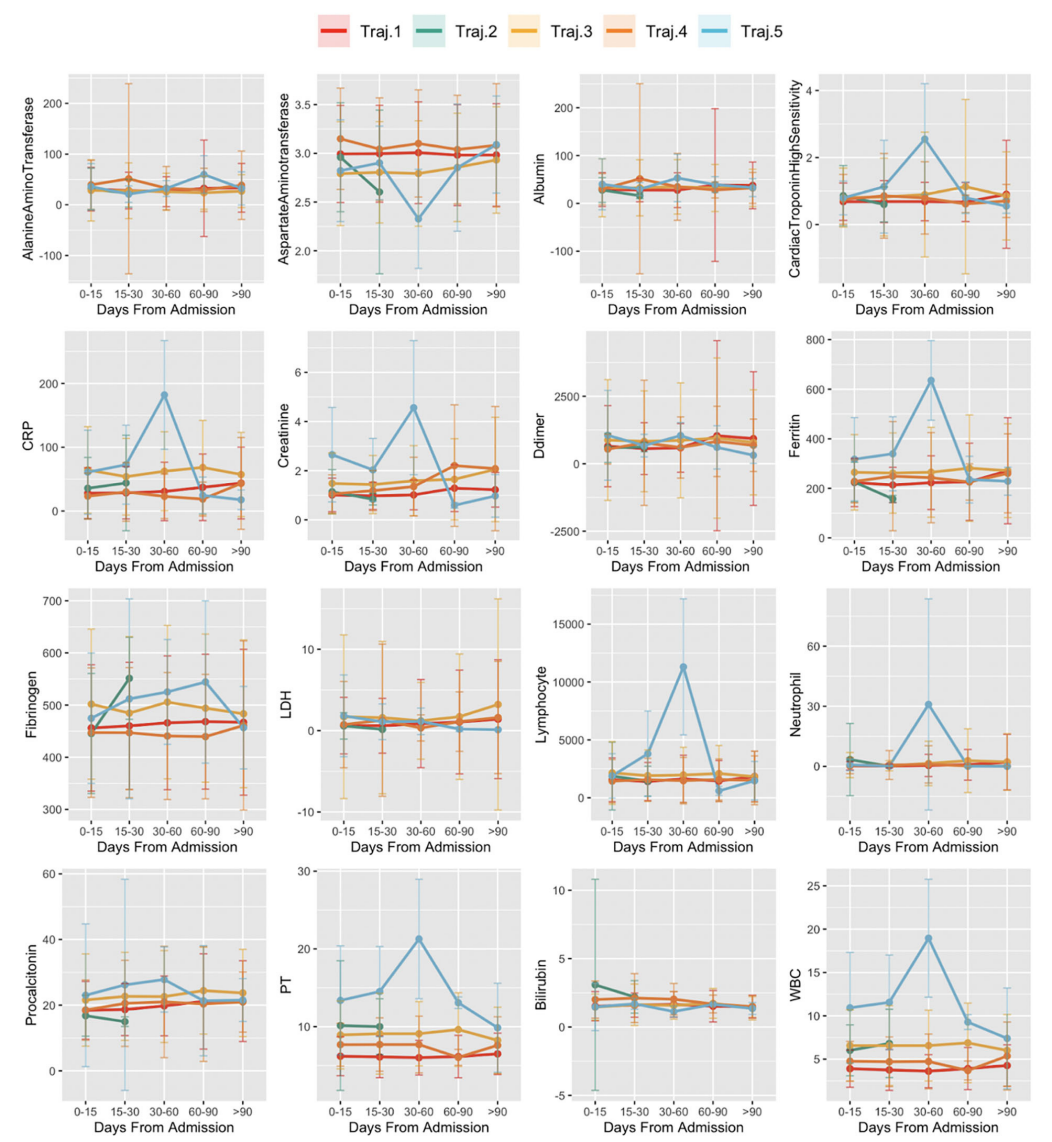
Laboratory values in time in trajectories. The x-axis indicates the disease stages time windows, from the first positive PCR test. Values of the y-axis indicate the average value of the laboratory test results in the time window and its standard error.

Figure 8 reports the distribution of laboratory test results over time of subjects by trajectory: x-axis indicates the disease stages time windows from the first positive PCR test. The y-axis indicates the average value of the laboratory test results in the time window and its standard error.

**Figure 9:**
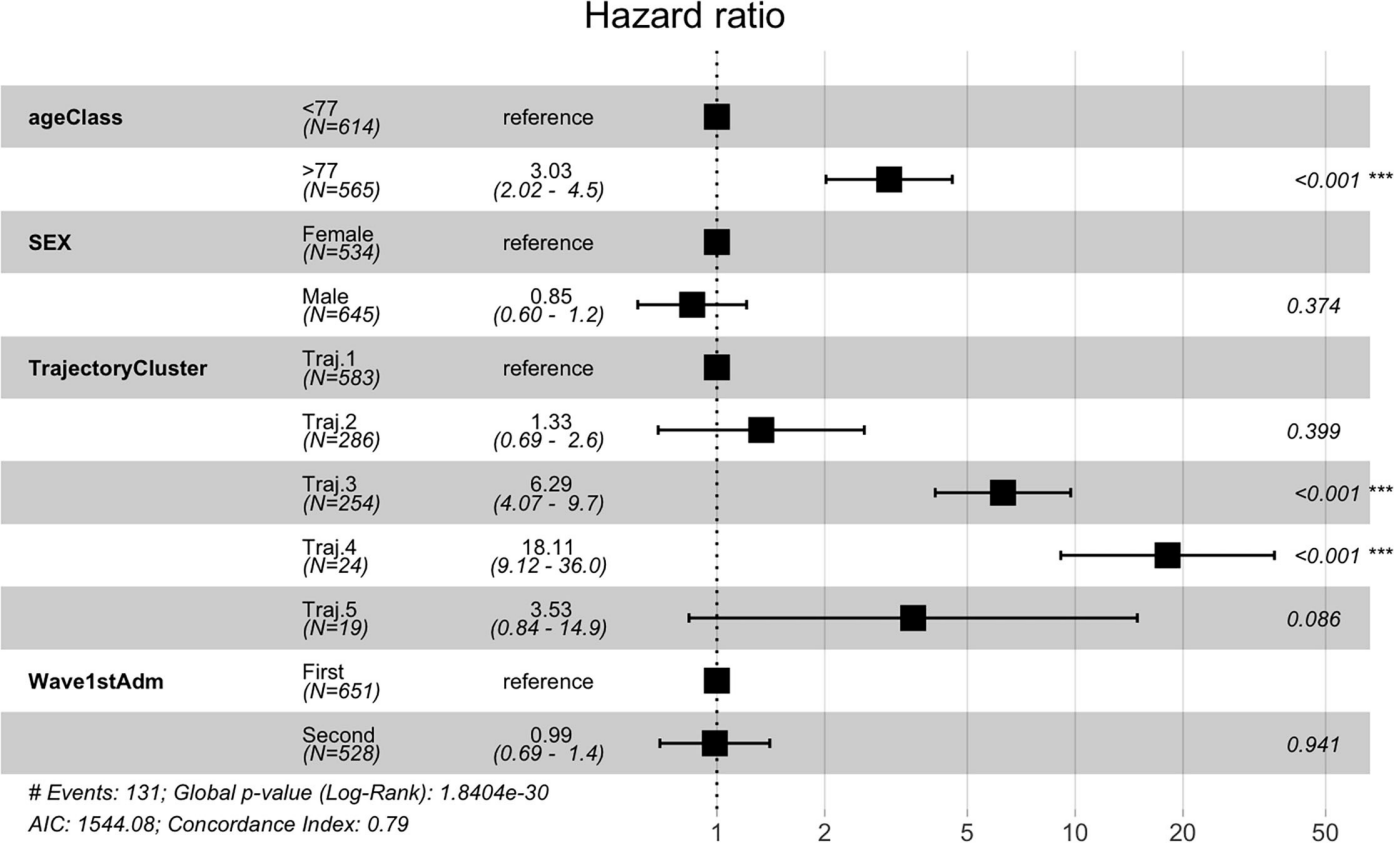
Cox Regression results reported as Hazard Ratio + 95 % confidence interval for hazard ratio and significance codes for death.

Temporal phenotypes were then compared in terms of survival. As indicated in Table 2, the groups with the worst prognosis are represented by Trajectory 3 and 4. We further investigated whether the phenotypes were significant predictors of death in the observation period by multivariate Cox proportional hazard regression including demographic and pandemic waves in the model. Trajectory 1 includes the majority of subjects and has been used as reference. Results from the multivariate regression are reported in Figure 9 and show that the mined temporal phenotypes are highly significant predictors of death: compared to subjects belonging to trajectory 1, subjects assigned to the phenotypes identified by trajectory 3 (HR = 6.29, 95% CI = 4.07 - 9.7, *p* < 2e-16) and trajectory 4 (HR = 18.11, 95% CI = 9.12 - 36.00, *p* < 2e-16) have a significantly higher risk of death. Complete results are in Supplementary Table 7.

### Mined Careflows and Comparison in Waves and Temporal Phenotypes

**Figure 10:**
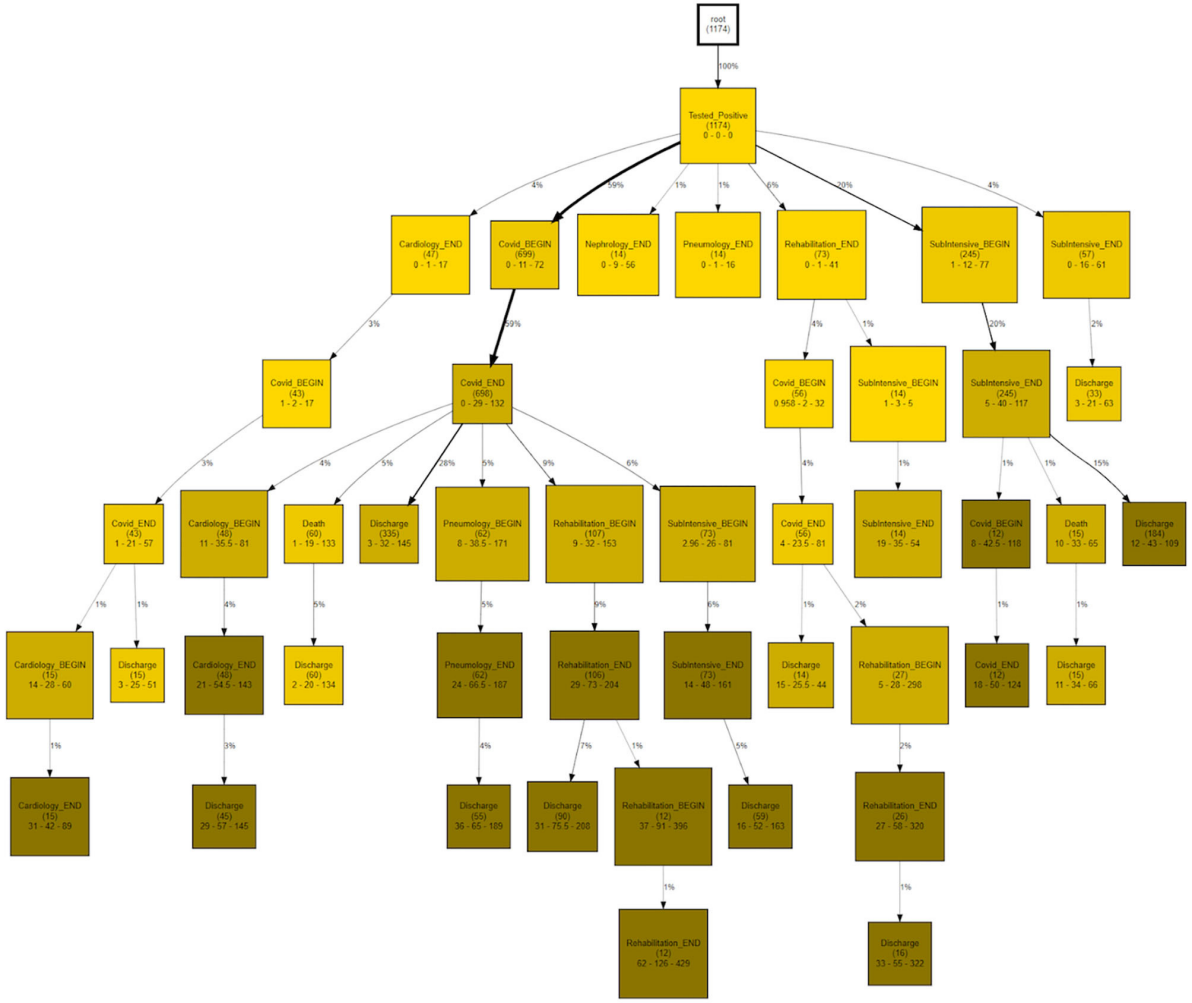
CareFlow miner results - time from the first event enrichment.

CareFlow Miner was exploited to dig into the data from many points of view. Figure 10 shows how the patients evolve, from an initial virtual state called root, through the events of their clinical pathways. Each node contains the name of the event (at the patient start with a positive covid test), the number of patients passing through that node (called hits) and a triple < t_min_ - t_med_ - t_max_ >) containing the minimum, the median and the maximum time took, from root, to reach the node. The colors depend on the median time and are shaded depending on the interval 0–10 (lightest); 10–25; 25–40; 40-Inf (darkest) days. On the edge is indicated the percentage of patients passing there on the basis of the number of patients in the previous node.

**Figure 11:**
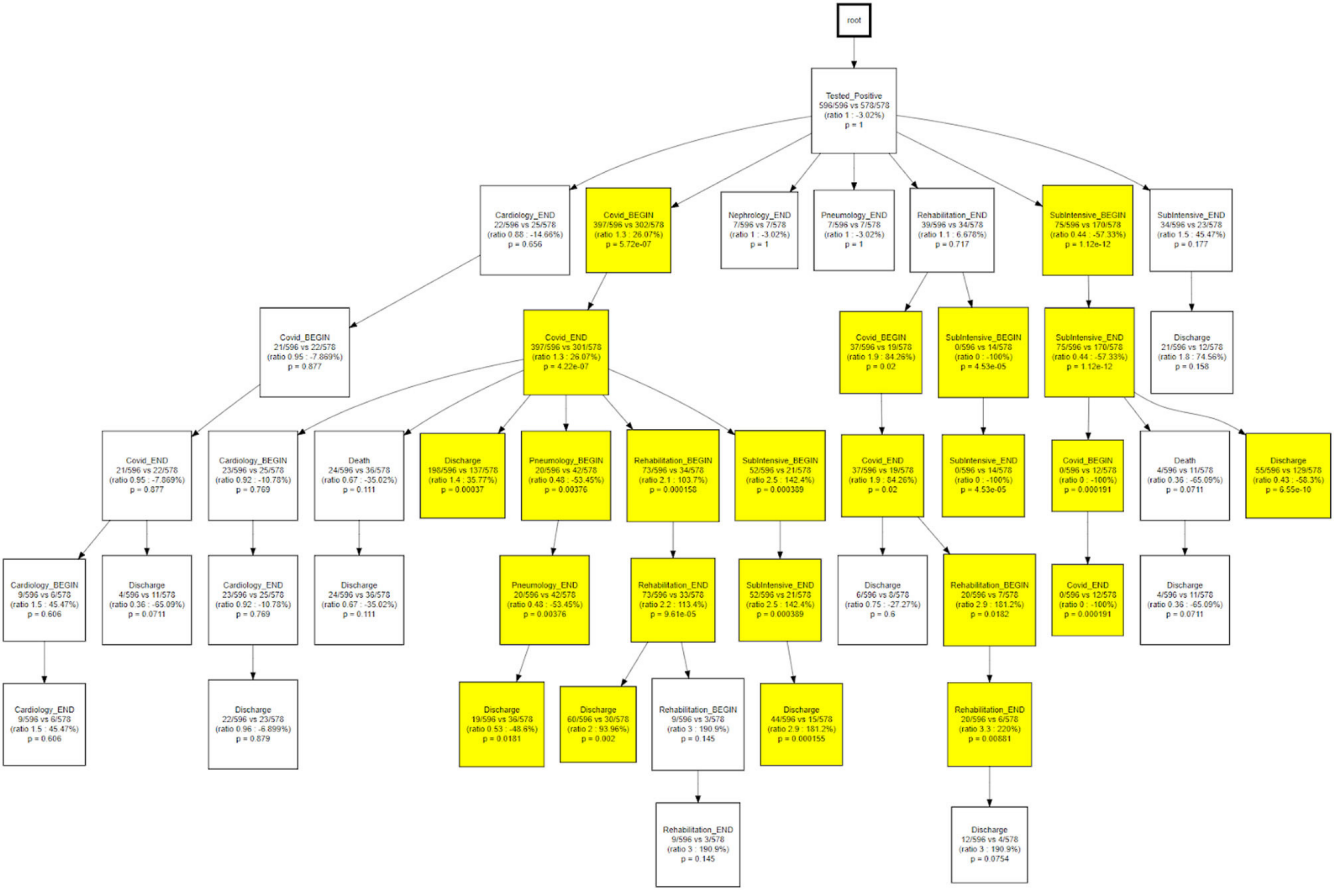
CareFlow miner results - waves comparison.

The same topology is used, in Figure 11, to investigate a different goal; in this case two different CareFlow Graphs were built splitting the data in two sub-cohorts, one for each wave. Each node contains, under the name of the event and for both the waves, the number of hits over the cardinality of the sub-cohort (1st wave vs 2nd wave). Below is indicated the ratio between the mentioned percentages and the *p*-value from the Fisher's exact test. By default, the library, indicated in yellow the nodes where the number of patients passing there between the two waves has *p*-value lower than 0.05 at statistical test.

As a final step, we compared the CareFlow among temporal phenotypes, applying the same approach used to compare waves. This step is aimed at combining process data (the health care processes mined via CFM) with physio-pathological information regarding the disease progression (the temporal phenotypes derived from the topological models).

**Figure 12:**
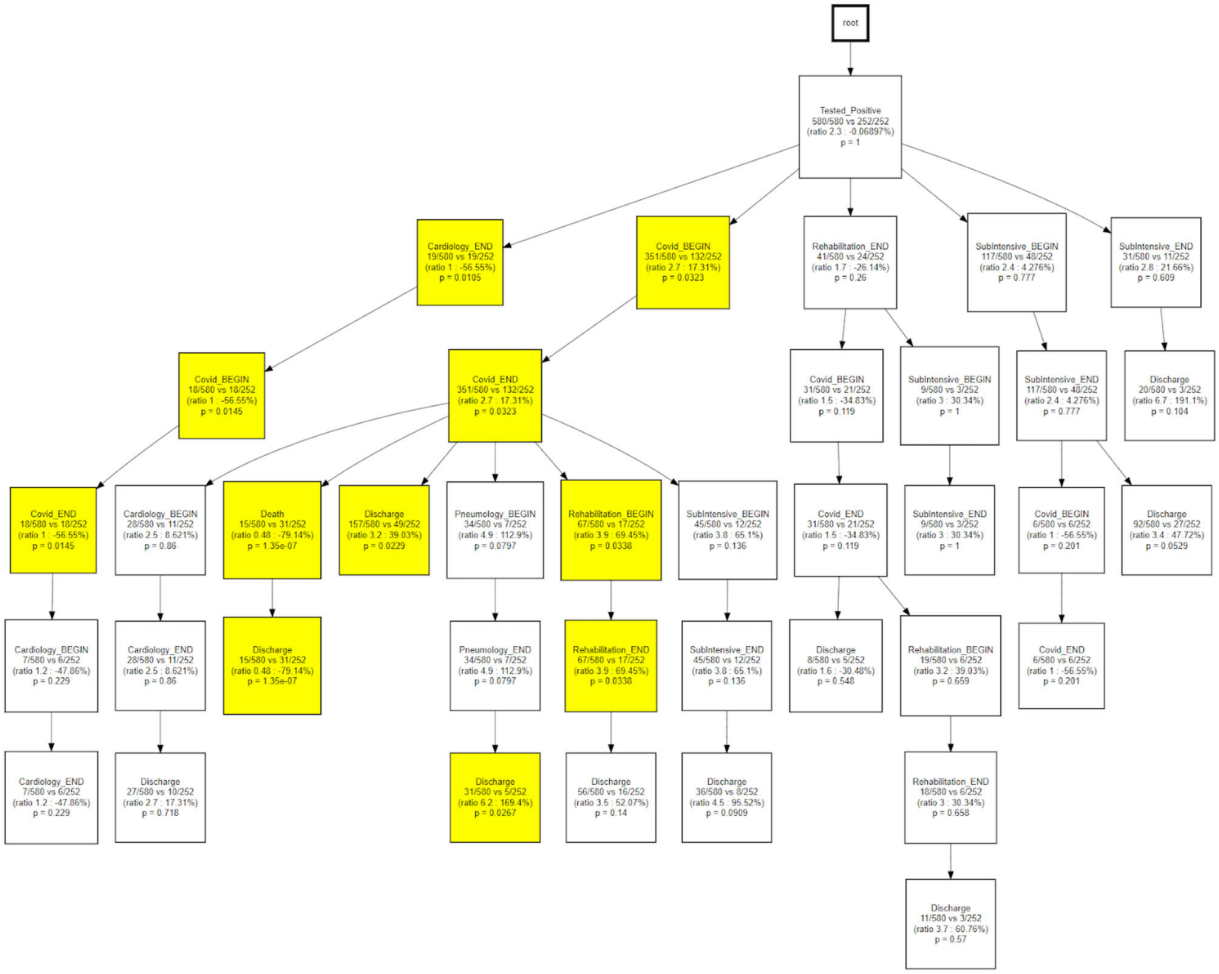
CareFlow miner results - comparison of trajectory 1 with trajectory 2.

Figure 12 shows how, when compared to the main trajectory 1, trajectory 2 identifies hospital processes with different outcomes following a hospitalization in the COVID wards, that is the probability of being discharged to home or the need of further treatments near a pneumatology unit.

**Figure 13:**
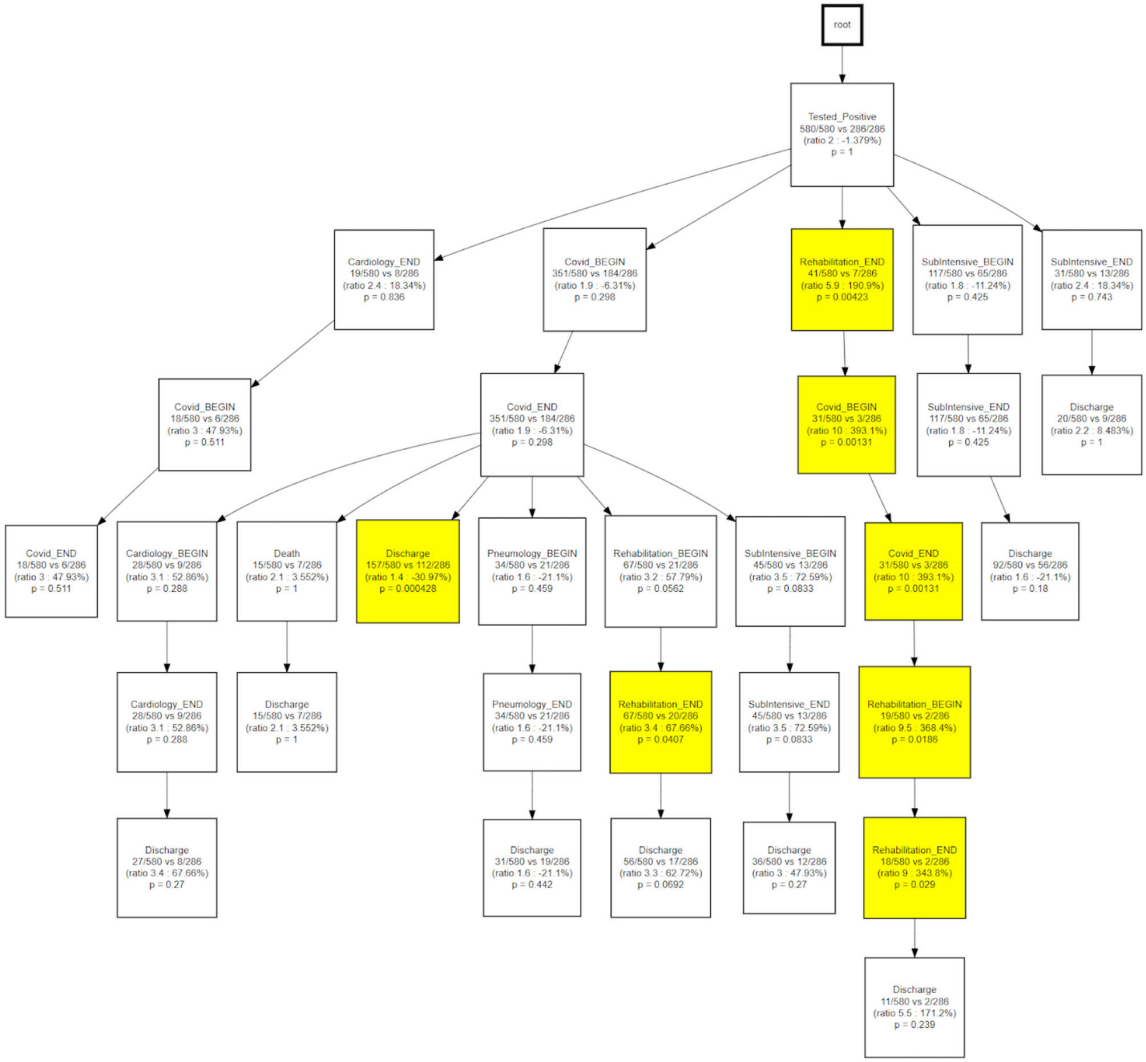
CareFlow miner results - comparison of trajectory 1 with trajectory 3.

Figure 13 indicates how trajectory 3 is able to differentiate the underlying status of patients following a rehabilitation care process.

## Discussion

The outbreak of the COVID-19 pandemic caused by the SARS-CoV-2 virus represents an unprecedented challenge for healthcare systems at a world-wide level. The first and most urgent aspect to be faced was represented by the management of a rapidly emerging and completely uncharacterized disease causing a dramatic rise in terms of mortality rate and secondary pathological conditions causing the collapse of most of the healthcare systems. Secondly, the conversion of entire hospital wards to COVID-19 dedicated units and the repurposing of physicians and nurses to assist COVID-19 patients induced an almost complete stop in terms of routine clinical activities (21). Thanks to the lessons learned and to the scientific knowledge gained during the first months of pandemic, national healthcare systems underwent an internal re-organizational phase that allowed facing COVID-19 disease in a more effective and harmonized way while allowing for the routine clinical activities to be recovered and performed in an almost physiological way (2, 22).

The extremely heterogeneous phenotypic manifestations characterizing COVID-19 patients (23) and the different long-term consequences of the infection (24) highlighted the need for personalized treatment interventions. To this aim it is of paramount importance to be able to identify potential subpopulations of patients characterized by similar disease manifestations with a common evolution over time. Further, being able to identify and characterize recurrent patterns of treatments and procedures and to verify the efficacy of such interventions against clinically relevant outcomes (e.g., severity, death) offers the possibility to improve patients' management during the acute and post-acute phases of the disease. A more effective management of COVID-19 patients would also translate into a lower burden on the healthcare systems, with shorter hospitalizations and lower treatment costs, toward an easier coexistence of both COVID-19 dedicated wards and regular units in the hospitals.

The availability of large amounts of retrospective EHR data of COVID-19 patients, analyzed by advanced data mining/artificial intelligence tools is providing an ever-increasing characterization of such phenotypic and management patterns, toward the ambitious goal of personalized medicine. The scientific articles recently published by the Consortium for Clinical Characterization of COVID-19 by EHR (4CE) [https://covidclinical.net/] (14, 25–27) confirm the usability of EHR data – consisting of demographic information, diagnoses, laboratory values, pharmacological treatments and interventions collected through hospitalizations and outpatient visits performed before, during and after COVID-19 disease – to successfully characterize COVID-19 related manifestations by different points of view.

The objective of this manuscript is to introduce useful tools to determine how specific patients' populations reflect into the healthcare dynamics of the hospital, to investigate how potential patients' subgroups respond to the different care processes and to generate novel hypotheses regarding the most effective healthcare strategies.

Available process mining tools typically have a purely descriptive connotation: quantitative and reproducible methodologies integrating aspects deriving from inferential statistics are therefore needed to be able to face real-world data challenges (10).

Furthermore, the Process Mining for Healthcare (PM4H) community recently described the process mining key challenges and especially the distinguishing characteristics that add to the complexity of using process mining within a healthcare context (28), including the need for tailored methodologies and frameworks, and the necessity to take into account concepts drifts given the changes of processes over time. These challenges have been partially tackled in the proposed framework, which mines the distinct change in pattern between the first and second wave of the pandemic, and identifies distinct disease trajectories, which has been recognized as an emerging area of study in process mining (29).

The analytical pipeline proposed in this manuscript consists of time series and process-oriented analyses, integrating visual analytics, inferential statistics with process mining approaches. The described methodology allows stratifying patients based on demographic and clinical characteristics as well as treatment and interventions performed during hospitalizations or outpatient visits. The described pipeline has been applied to real-world data deriving from COVID-19 patients hospitalized in three ICSM centers in Lombardy region, Italy.

Descriptive statistics allowed characterizing the analyzed cohort of subjects based on demographics characteristics, including age, gender and ICSM hospitalization center. By stratifying subjects according to the calendar date when the SARS-CoV-2 infection first occurred it has been also possible to analyze and compare patients' characteristics between COVID-19 pandemic waves. Disease categories prevalence rates have been also estimated on the basis of ICD9 codes as function of different time windows, using the SARS-CoV-2 positive test as reference (pre, acute, post and long). Such categories showed differential trends as function of the stage, on the basis of patients' age. Circulatory system was the most represented category, with a higher frequency in older people compared to younger people, especially during pre and acute phases. Patient's hospitalizations distributions have been compared between wards as function of age, gender, waves and center, showing differential patterns, especially between centers. Such differences could be partially explained by the characteristics of the ICSM centers and by the number of wards converted into COVID-19 units to face the pandemic pressure.

Survival analyses allowed identifying statistically significant differences in terms of survival profiles between first and second wave, with patients from the second wave being characterized by a significantly higher survival probability. As previously reported, younger patients were also more likely to survive compared to older subjects (30).

Temporal disease trajectories are usually constructed on the basis of discrete events (i.e., diagnosis) and defined as an ordered series of diagnoses where the diagnoses were observed in the patient in a specific order, then infer the latent disease stages from that (31). In this work the TDA approach, coupled with the concept of pseudo time (20). The TDA approach is “censoring agnostic,” based on continuous observations (i.e., lab values) and reconstructs the trajectory on the basis of observed disease stages with the pace of intra-patient follow-ups. On the contrary, the CFM approach sets an initial time - in this application the initial time is the first positive test that we use as a diagnosis proxy - and mines processes in a sequential way from discrete events. When we compare CFM mined processes in terms of TDA trajectories (Figures 12, 13) we are merging these two timelines perspectives: the continuous one from the TDA and the discrete one from the CFM. Figures 12, 13 can be interpreted together with the patients' characteristic in each trajectory (i.e., Table 2), indicating how population strata, identified as temporal phenotypes, differ from the physiological point of view and also reflects how patients' fragility is managed during the care process.

The TDA algorithm identified five potential trajectories, from the laboratory values registered during patients' acute hospitalizations and follow-ups. Using baseline information, it has been possible to characterize patients belonging to the trajectories most represented in terms of sample size. Significant differences among trajectories were observed in terms of age at first test, death, Charlson Comorbidity Index at Admission but not in pandemic waves. Patients from different trajectories showed different trends in terms of lab values as function of the time from the first positive SARS-CoV-2 swab test, being also characterized by statistically significant different death risk estimates.

The CareFlow Miner tool was exploited to study how patients evolve over time - from an initial virtual state, through the events characterizing their clinical pathways. Thanks to a tree-like graphical structure (the CareFlow Graph) it is possible to inspect the characteristics of the different paths defined by sequential nodes. Different CareFlow Graphs were built splitting the data in sub-cohorts as function of the pandemic waves and of the temporal trajectories showing statistically significant differences in terms of nodes characteristics.

This study has several limitations. Further analyses should be done to better describe intra-waves dynamics with finer granularity. The inclusion of data from the third wave, with additional information regarding variants and vaccination could strengthen the presented results.

Given the recent application of the proposed method in healthcare to heterogeneous clinical and administrative data, they still have several limitations. Further efforts might be needed to better visualize CFM results, for example not coloring nodes in a binary way according to fixed threshold but reporting the *p*-values as a continuous scale. However, we feel that the current visualization is the best (simplest) one to explaining the major differences among the considered strata in purely descriptive fashion. These tools still need to be introduced into clinical practice and/or delivered to public health stockholders, which implies their practical evaluation, thoughtful interpretation and usefulness assessment, for example for their embedding in decision support systems. While their potential impact on routine clinical practice has been already shown (32), we still have to prove their validity in emergency contexts such as the outbreak of the COVID-19 pandemic.

## Data Availability Statement

The data analyzed in this study is subject to the following licenses/restrictions: The datasets generated for this study are available on request to the corresponding author as aggregated data. Requests to access these datasets should be directed to arianna.dagliati@unipv.it.

## Ethics Statement

The studies involving human participants were reviewed and approved by 2518 CE of the Istituti Clinici Scientifici Maugeri. Written informed consent for participation was not required for this study in accordance with the national legislation and the institutional requirements.

## Author Contributions

AD, RG, AM, VT, LS, FC, and RB: concept. AD and RG: analysis execution. VT and AM: data extraction and pre-processing. AD, RG, AM, and RB: initial writing. LC, FC, and RB: draft revision. All authors have contributed to the design and assessment of results of the study as well as read and approved the manuscript.

## Conflict of Interest

The authors declare that the research was conducted in the absence of any commercial or financial relationships that could be construed as a potential conflict of interest.

## Publisher's Note

All claims expressed in this article are solely those of the authors and do not necessarily represent those of their affiliated organizations, or those of the publisher, the editors and the reviewers. Any product that may be evaluated in this article, or claim that may be made by its manufacturer, is not guaranteed or endorsed by the publisher.
