# Supplementary material for: A Process Mining Pipeline to Characterize COVID-19 Patients' Trajectories and Identify Relevant Temporal Phenotypes From EHR Data
Source: Front Public Health. 2022 May 23;10:815674. doi: 10.3389/fpubh.2022.815674 (PMC9168006; doi:10.3389/fpubh.2022.815674)

***Supplementary Figures and Tables***

TABLE 1s - Number of patients in quarters (first hospital admission)

| **Year quarter** | **Num. patients** |
| --- | --- |
| 2020.1 | 285 |
| 2020.2 | 329 |
| 2020.3 | 26 |
| 2020.4 | 362 |
| 2021.1 | 177 |

TABLE 2s - PheCodes related to respiratory infections, as excluded for study patients comorbidities.

| **PheCode Category** | **First Wave** | **Second Wave** |
| --- | --- | --- |
| COVID-19 | 105 | 40 |
| infectious diseases | 1 | 2 |
| respiratory | 59 | 30 |

| **Phe Codes** | **First Wave** | **Second Wave** |
| --- | --- | --- |
| Allergic rhinitis | 1 | 0 |
| Asthma | 1 | 0 |
| Bacterial infection NOS | 0 | 2 |
| Chronic airway obstruction | 1 | 8 |
| Intestinal infection | 0 | 2 |
| Lung disease due to external agents | 0 | 1 |
| Other alveolar and parietoalveolar pneumonopathy | 2 | 0 |
| Other diseases of respiratory system, not elsewhere classified | 3 | 0 |
| Other infectious and parasitic diseases | 1 | 0 |
| Pneumonia | 91 | 2 |
| Postinflammatory pulmonary fibrosis | 0 | 2 |
| Respiratory failure, insufficiency, arrest | 50 | 18 |
| Viral infection | 15 | 3 |

| **Phe Codes** | **pre stage** | **acute stage** | **post stage** |
| --- | --- | --- | --- |
| Allergic rhinitis | 0 | 1 | 0 |
| Asthma | 0 | 0 | 1 |
| Bacterial infection NOS | 0 | 1 | 1 |
| Chronic airway obstruction | 2 | 7 | 0 |
| Intestinal infection | 0 | 1 | 1 |
| Lung disease due to external agents | 0 | 0 | 1 |
| Other alveolar and parietoalveolar pneumonopathy | 0 | 1 | 1 |
| Other diseases of respiratory system, not elsewhere classified | 0 | 2 | 1 |
| Other infectious and parasitic diseases | 0 | 0 | 1 |
| Pneumonia | 3 | 79 | 11 |
| Postinflammatory pulmonary fibrosis | 0 | 1 | 1 |
| Respiratory failure, insufficiency, arrest | 2 | 53 | 13 |
| Viral infection | 1 | 17 | 0 |

TABLE 3s - Patients distribution in yearly quarters, stratified by age, sex a hospital

| **Age Class** | **Quarter** | **Number of Subject** | **Perc** |
| --- | --- | --- | --- |
| 69_77 | 2020.1 | 58,00 | 24,37% |
| 78_84 | 2020.1 | 38,00 | 15,97% |
| mag_84 | 2020.1 | 54,00 | 22,69% |
| min_68 | 2020.1 | 88,00 | 36,97% |
| 69_77 | 2020.2 | 106,00 | 24,26% |
| 78_84 | 2020.2 | 84,00 | 19,22% |
| mag_84 | 2020.2 | 94,00 | 21,51% |
| min_68 | 2020.2 | 153,00 | 35,01% |
| 69_77 | 2020.3 | 11,00 | 24,44% |
| 78_84 | 2020.3 | 8,00 | 17,78% |
| mag_84 | 2020.3 | 8,00 | 17,78% |
| min_68 | 2020.3 | 18,00 | 40,00% |
| 69_77 | 2020.4 | 94,00 | 22,87% |
| 78_84 | 2020.4 | 105,00 | 25,55% |
| mag_84 | 2020.4 | 111,00 | 27,01% |
| min_68 | 2020.4 | 101,00 | 24,57% |
| 69_77 | 2021.1 | 46,00 | 19,74% |
| 78_84 | 2021.1 | 67,00 | 28,76% |
| mag_84 | 2021.1 | 72,00 | 30,90% |
| min_68 | 2021.1 | 48,00 | 20,60% |
| 69_77 | 2021.2 | 5,00 | 45,45% |
| 78_84 | 2021.2 | 2,00 | 18,18% |
| mag_84 | 2021.2 | 1,00 | 9,09% |
| min_68 | 2021.2 | 3,00 | 27,27% |
| 69_77 | 2021.3 | 3,00 | 50,00% |
| 78_84 | 2021.3 | 2,00 | 33,33% |
| min_68 | 2021.3 | 1,00 | 16,67% |

| **SEX** | **Quarter** | **Number of Subject** | **Perc** |
| --- | --- | --- | --- |
| F | 2020.1 | 107,00 | 44,96% |
| F | 2020.2 | 174,00 | 39,82% |
| F | 2020.3 | 23,00 | 51,11% |
| F | 2020.4 | 204,00 | 49,64% |
| F | 2021.1 | 102,00 | 43,78% |
| F | 2021.2 | 4,00 | 36,36% |
| F | 2021.3 | 2,00 | 33,33% |
| M | 2020.1 | 131,00 | 55,04% |
| M | 2020.2 | 263,00 | 60,18% |
| M | 2020.3 | 22,00 | 48,89% |
| M | 2020.4 | 207,00 | 50,36% |
| M | 2021.1 | 131,00 | 56,22% |
| M | 2021.2 | 7,00 | 63,64% |
| M | 2021.3 | 4,00 | 66,67% |

| **Centre** | **Quarter** | **Number of Subject** | **Perc** |
| --- | --- | --- | --- |
| Lumezzane | 2020.1 | 82,00 | 34,45% |
| Lumezzane | 2020.2 | 106,00 | 24,26% |
| Lumezzane | 2020.3 | 7,00 | 15,56% |
| Lumezzane | 2020.4 | 76,00 | 18,49% |
| Lumezzane | 2021.1 | 84,00 | 36,05% |
| Lumezzane | 2021.2 | 1,00 | 9,09% |
| Lumezzane | 2021.3 | 1,00 | 16,67% |
| Milano Camaldoli | 2020.1 | 50,00 | 21,01% |
| Milano Camaldoli | 2020.2 | 115,00 | 26,32% |
| Milano Camaldoli | 2020.3 | 16,00 | 35,56% |
| Milano Camaldoli | 2020.4 | 131,00 | 31,87% |
| Milano Camaldoli | 2021.1 | 89,00 | 38,20% |
| Milano Camaldoli | 2021.2 | 1,00 | 9,09% |
| Pavia | 2020.1 | 106,00 | 44,54% |
| Pavia | 2020.2 | 216,00 | 49,43% |
| Pavia | 2020.3 | 22,00 | 48,89% |
| Pavia | 2020.4 | 204,00 | 49,64% |
| Pavia | 2021.1 | 60,00 | 25,75% |
| Pavia | 2021.2 | 9,00 | 81,82% |
| Pavia | 2021.3 | 5,00 | 83,33% |

TABLE 5s - PheCodes diagnostic categories distribution in disease stage and age class.

| **category** | **stage** | **ageClass** | **Number** | **Number in Stage** | **Prevalence in Stage** |
| --- | --- | --- | --- | --- | --- |
| circulatory system | acute | mag_77 | 272,00 | 867,00 | 31,37% |
| circulatory system | post | mag_77 | 38,00 | 185,00 | 20,54% |
| circulatory system | pre | mag_77 | 100,00 | 321,00 | 31,15% |
| circulatory system | acute | min_77 | 180,00 | 643,00 | 27,99% |
| circulatory system | post | min_77 | 58,00 | 265,00 | 21,89% |
| circulatory system | pre | min_77 | 112,00 | 423,00 | 26,48% |
| congenital anomalies | acute | mag_77 | 2,00 | 867,00 | 0,23% |
| congenital anomalies | pre | mag_77 | 1,00 | 321,00 | 0,31% |
| congenital anomalies | acute | min_77 | 3,00 | 643,00 | 0,47% |
| congenital anomalies | pre | min_77 | 4,00 | 423,00 | 0,95% |
| dermatologic | acute | mag_77 | 10,00 | 867,00 | 1,15% |
| dermatologic | long | mag_77 | 1,00 | 8,00 | 12,50% |
| dermatologic | post | mag_77 | 2,00 | 185,00 | 1,08% |
| dermatologic | pre | mag_77 | 3,00 | 321,00 | 0,93% |
| dermatologic | acute | min_77 | 12,00 | 643,00 | 1,87% |
| dermatologic | post | min_77 | 9,00 | 265,00 | 3,40% |
| dermatologic | pre | min_77 | 9,00 | 423,00 | 2,13% |
| digestive | acute | mag_77 | 32,00 | 867,00 | 3,69% |
| digestive | post | mag_77 | 12,00 | 185,00 | 6,49% |
| digestive | pre | mag_77 | 10,00 | 321,00 | 3,12% |
| digestive | acute | min_77 | 33,00 | 643,00 | 5,13% |
| digestive | long | min_77 | 4,00 | 16,00 | 25,00% |
| digestive | post | min_77 | 12,00 | 265,00 | 4,53% |
| digestive | pre | min_77 | 23,00 | 423,00 | 5,44% |
| endocrine/metabolic | acute | mag_77 | 145,00 | 867,00 | 16,72% |
| endocrine/metabolic | long | mag_77 | 1,00 | 8,00 | 12,50% |
| endocrine/metabolic | post | mag_77 | 27,00 | 185,00 | 14,59% |
| endocrine/metabolic | pre | mag_77 | 35,00 | 321,00 | 10,90% |
| endocrine/metabolic | acute | min_77 | 129,00 | 643,00 | 20,06% |
| endocrine/metabolic | long | min_77 | 2,00 | 16,00 | 12,50% |
| endocrine/metabolic | post | min_77 | 39,00 | 265,00 | 14,72% |
| endocrine/metabolic | pre | min_77 | 51,00 | 423,00 | 12,06% |
| genitourinary | acute | mag_77 | 86,00 | 867,00 | 9,92% |
| genitourinary | long | mag_77 | 1,00 | 8,00 | 12,50% |
| genitourinary | post | mag_77 | 19,00 | 185,00 | 10,27% |
| genitourinary | pre | mag_77 | 47,00 | 321,00 | 14,64% |
| genitourinary | acute | min_77 | 51,00 | 643,00 | 7,93% |
| genitourinary | long | min_77 | 1,00 | 16,00 | 6,25% |
| genitourinary | post | min_77 | 27,00 | 265,00 | 10,19% |
| genitourinary | pre | min_77 | 49,00 | 423,00 | 11,58% |
| hematopoietic | acute | mag_77 | 71,00 | 867,00 | 8,19% |
| hematopoietic | long | mag_77 | 1,00 | 8,00 | 12,50% |
| hematopoietic | post | mag_77 | 16,00 | 185,00 | 8,65% |
| hematopoietic | pre | mag_77 | 16,00 | 321,00 | 4,98% |
| hematopoietic | acute | min_77 | 31,00 | 643,00 | 4,82% |
| hematopoietic | long | min_77 | 2,00 | 16,00 | 12,50% |
| hematopoietic | post | min_77 | 22,00 | 265,00 | 8,30% |
| hematopoietic | pre | min_77 | 26,00 | 423,00 | 6,15% |
| injuries & poisonings | acute | mag_77 | 44,00 | 867,00 | 5,07% |
| injuries & poisonings | long | mag_77 | 2,00 | 8,00 | 25,00% |
| injuries & poisonings | post | mag_77 | 15,00 | 185,00 | 8,11% |
| injuries & poisonings | pre | mag_77 | 32,00 | 321,00 | 9,97% |
| injuries & poisonings | acute | min_77 | 27,00 | 643,00 | 4,20% |
| injuries & poisonings | long | min_77 | 3,00 | 16,00 | 18,75% |
| injuries & poisonings | post | min_77 | 7,00 | 265,00 | 2,64% |
| injuries & poisonings | pre | min_77 | 36,00 | 423,00 | 8,51% |
| mental disorders | acute | mag_77 | 51,00 | 867,00 | 5,88% |
| mental disorders | post | mag_77 | 8,00 | 185,00 | 4,32% |
| mental disorders | pre | mag_77 | 9,00 | 321,00 | 2,80% |
| mental disorders | acute | min_77 | 38,00 | 643,00 | 5,91% |
| mental disorders | post | min_77 | 16,00 | 265,00 | 6,04% |
| mental disorders | pre | min_77 | 13,00 | 423,00 | 3,07% |
| musculoskeletal | acute | mag_77 | 28,00 | 867,00 | 3,23% |
| musculoskeletal | post | mag_77 | 4,00 | 185,00 | 2,16% |
| musculoskeletal | pre | mag_77 | 11,00 | 321,00 | 3,43% |
| musculoskeletal | acute | min_77 | 27,00 | 643,00 | 4,20% |
| musculoskeletal | long | min_77 | 1,00 | 16,00 | 6,25% |
| musculoskeletal | post | min_77 | 8,00 | 265,00 | 3,02% |
| musculoskeletal | pre | min_77 | 7,00 | 423,00 | 1,65% |
| neoplasms | acute | mag_77 | 31,00 | 867,00 | 3,58% |
| neoplasms | post | mag_77 | 10,00 | 185,00 | 5,41% |
| neoplasms | pre | mag_77 | 10,00 | 321,00 | 3,12% |
| neoplasms | acute | min_77 | 34,00 | 643,00 | 5,29% |
| neoplasms | long | min_77 | 2,00 | 16,00 | 12,50% |
| neoplasms | post | min_77 | 11,00 | 265,00 | 4,15% |
| neoplasms | pre | min_77 | 23,00 | 423,00 | 5,44% |
| neurological | acute | mag_77 | 69,00 | 867,00 | 7,96% |
| neurological | long | mag_77 | 1,00 | 8,00 | 12,50% |
| neurological | post | mag_77 | 22,00 | 185,00 | 11,89% |
| neurological | pre | mag_77 | 37,00 | 321,00 | 11,53% |
| neurological | acute | min_77 | 53,00 | 643,00 | 8,24% |
| neurological | long | min_77 | 1,00 | 16,00 | 6,25% |
| neurological | post | min_77 | 40,00 | 265,00 | 15,09% |
| neurological | pre | min_77 | 58,00 | 423,00 | 13,71% |
| pregnancy complications | pre | mag_77 | 3,00 | 321,00 | 0,93% |
| pregnancy complications | acute | min_77 | 3,00 | 643,00 | 0,47% |
| pregnancy complications | pre | min_77 | 5,00 | 423,00 | 1,18% |
| sense organs | acute | mag_77 | 10,00 | 867,00 | 1,15% |
| sense organs | post | mag_77 | 1,00 | 185,00 | 0,54% |
| sense organs | pre | mag_77 | 2,00 | 321,00 | 0,62% |
| sense organs | acute | min_77 | 8,00 | 643,00 | 1,24% |
| sense organs | pre | min_77 | 2,00 | 423,00 | 0,47% |
| symptoms | acute | mag_77 | 16,00 | 867,00 | 1,85% |
| symptoms | long | mag_77 | 1,00 | 8,00 | 12,50% |
| symptoms | post | mag_77 | 11,00 | 185,00 | 5,95% |
| symptoms | pre | mag_77 | 5,00 | 321,00 | 1,56% |
| symptoms | acute | min_77 | 14,00 | 643,00 | 2,18% |
| symptoms | post | min_77 | 16,00 | 265,00 | 6,04% |
| symptoms | pre | min_77 | 5,00 | 423,00 | 1,18% |

TABLE 6s - Events Counts

| **Event** | **Number of Event** |
| --- | --- |
| Surgery | 17 |
| Oncology | 29 |
| Nephrology | 45 |
| ICU | 53 |
| Pneumology | 138 |
| Cardiology | 185 |
| Rehabilitation | 386 |
| SubIntensive | 480 |
| Access To Hospital | 596 |
| Covid | 899 |
| Discharge | 1169 |
| Tested Positive | 1179 |

Table 7s - Cox Regression Results

Call:

coxph(formula = Surv(Time, DEATH) ~ ageClass + SEX + TrajectoryCluster +

Wave1stAdm, data = CoxData)

coef exp(coef) se(coef) z p

ageClass>77 1.10695 3.02513 0.20690 5.350 8.78e-08

SEXMale -0.15882 0.85315 0.17858 -0.889 0.3738

TrajectoryClusterTraj.2 0.28514 1.32995 0.33792 0.844 0.3988

TrajectoryClusterTraj.3 1.83858 6.28760 0.22212 8.277 < 2e-16

TrajectoryClusterTraj.4 2.89653 18.11118 0.35021 8.271 < 2e-16

TrajectoryClusterTraj.5 1.26120 3.52965 0.73503 1.716 0.0862

Wave1stAdmSecond -0.01325 0.98683 0.18017 -0.074 0.9414

Likelihood ratio test=156.4 on 7 df, p=< 2.2e-16

n= 1166, number of events= 131

(13 observations deleted due to missingness)

***Supplementary Analyses for Test Assumptions and Models Validation***

**Age Distributions and test assumptions**


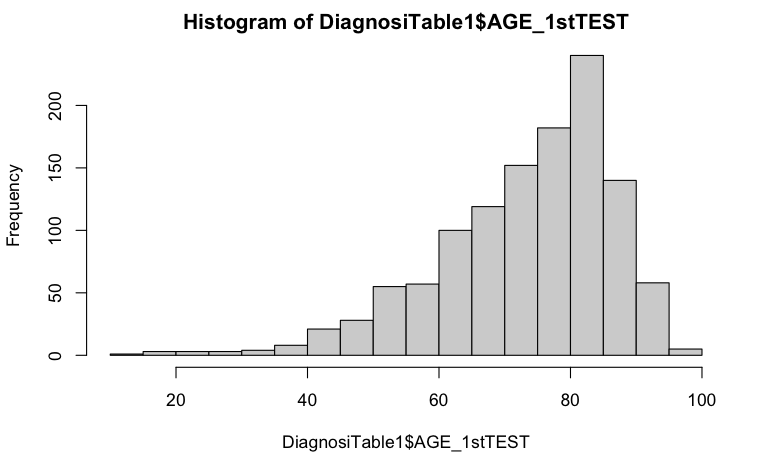


Shapiro Test Results

data: DiagnosiTable1$AGE_1stTEST

W = 0.93795, p-value < 2.2e-16

Age Distributions in Waves
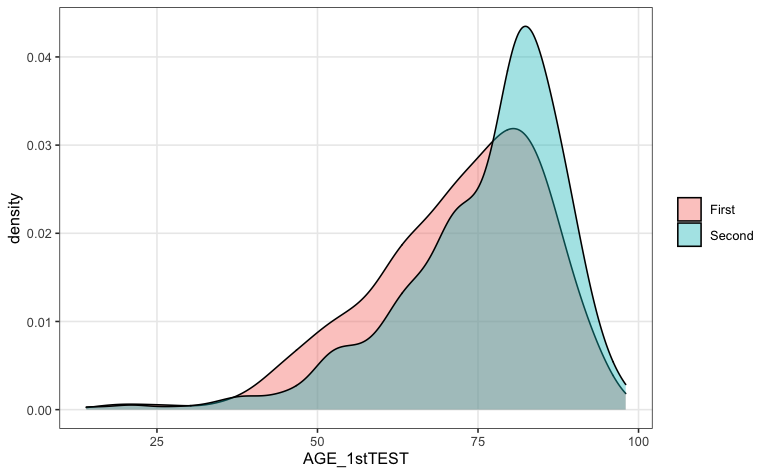


Age Distribution in Trajectories


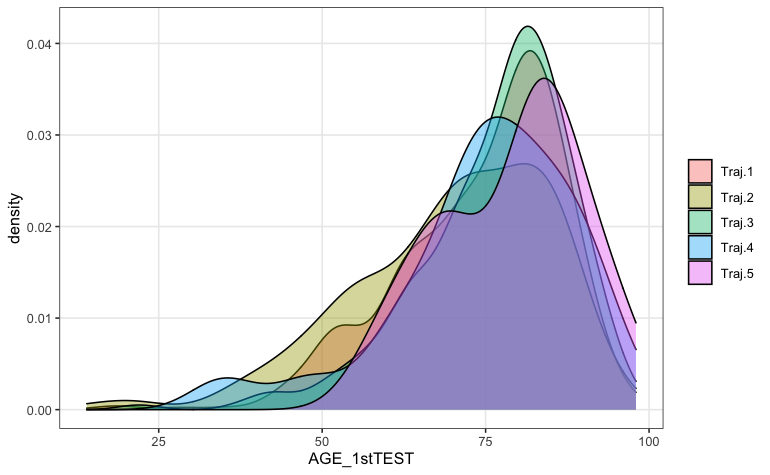


# Charlson Score Distribution and test assumptions


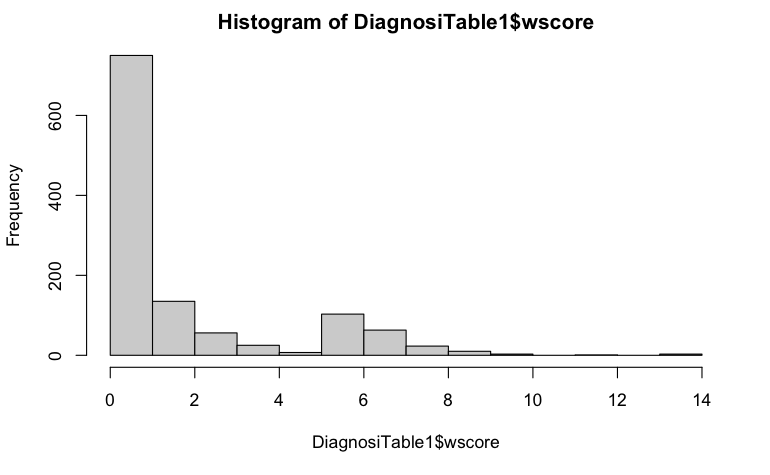


Shapiro Test Results

data: DiagnosiTable1$wscore

W = 0.74963, p-value < 2.2e-16

Charlson Score Distribution in Waves


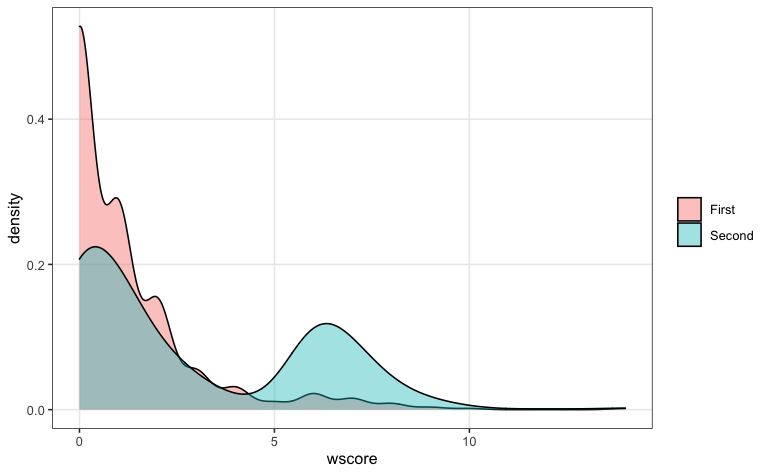


Charlson Score Distribution in Trajectories


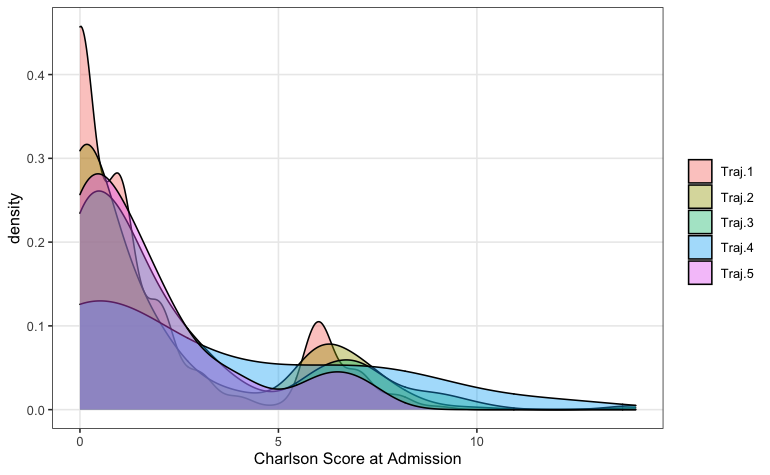


**Cox Regression model validation**

proportional-hazards (PH) assumption

chisq df p

ageClass 1.33834 1 0.247

SEX 0.00295 1 0.957

TrajectoryCluster 8.94816 4 0.062

Wave1stAdm 1.63421 1 0.201

GLOBAL 12.73466 7 0.079


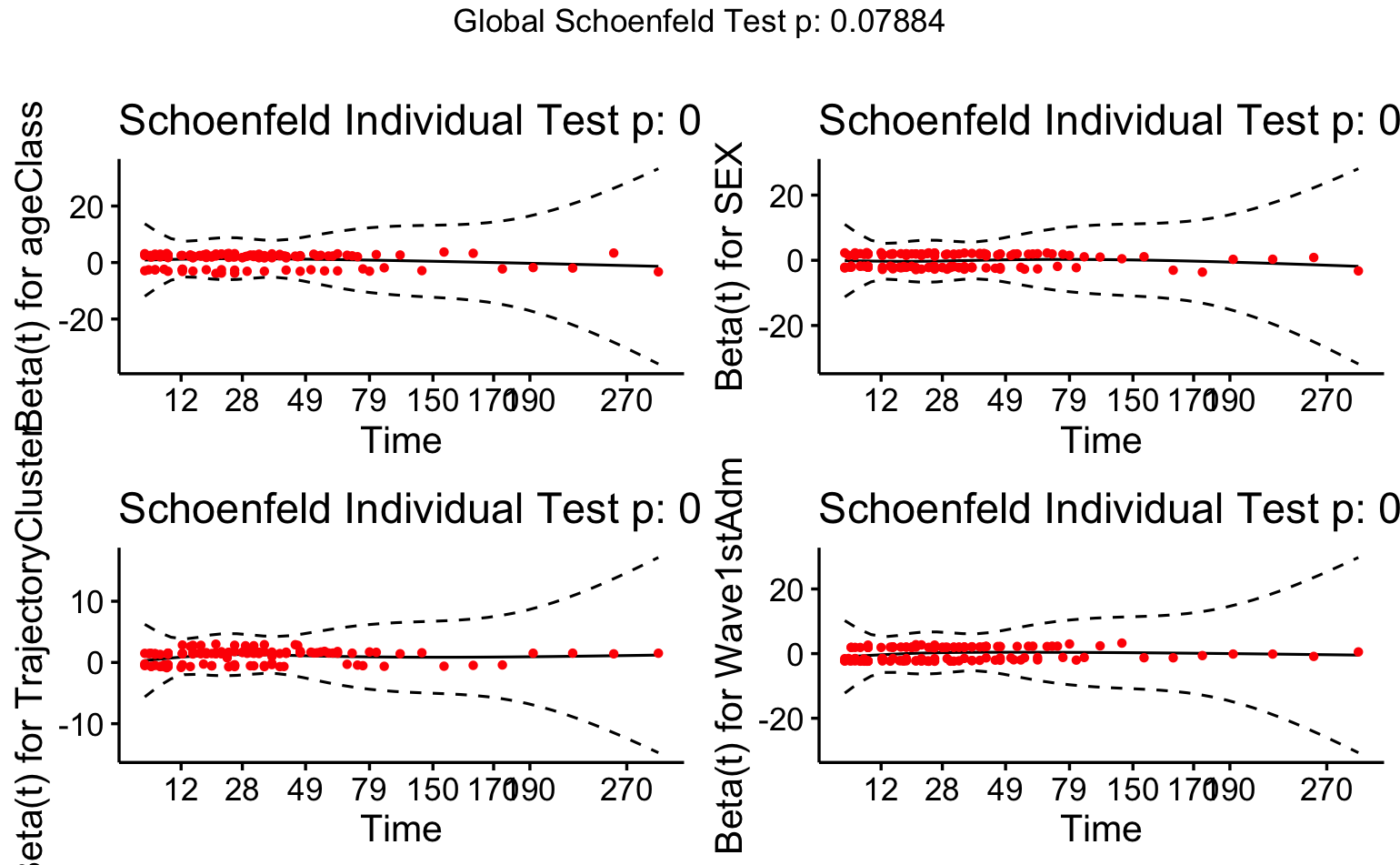


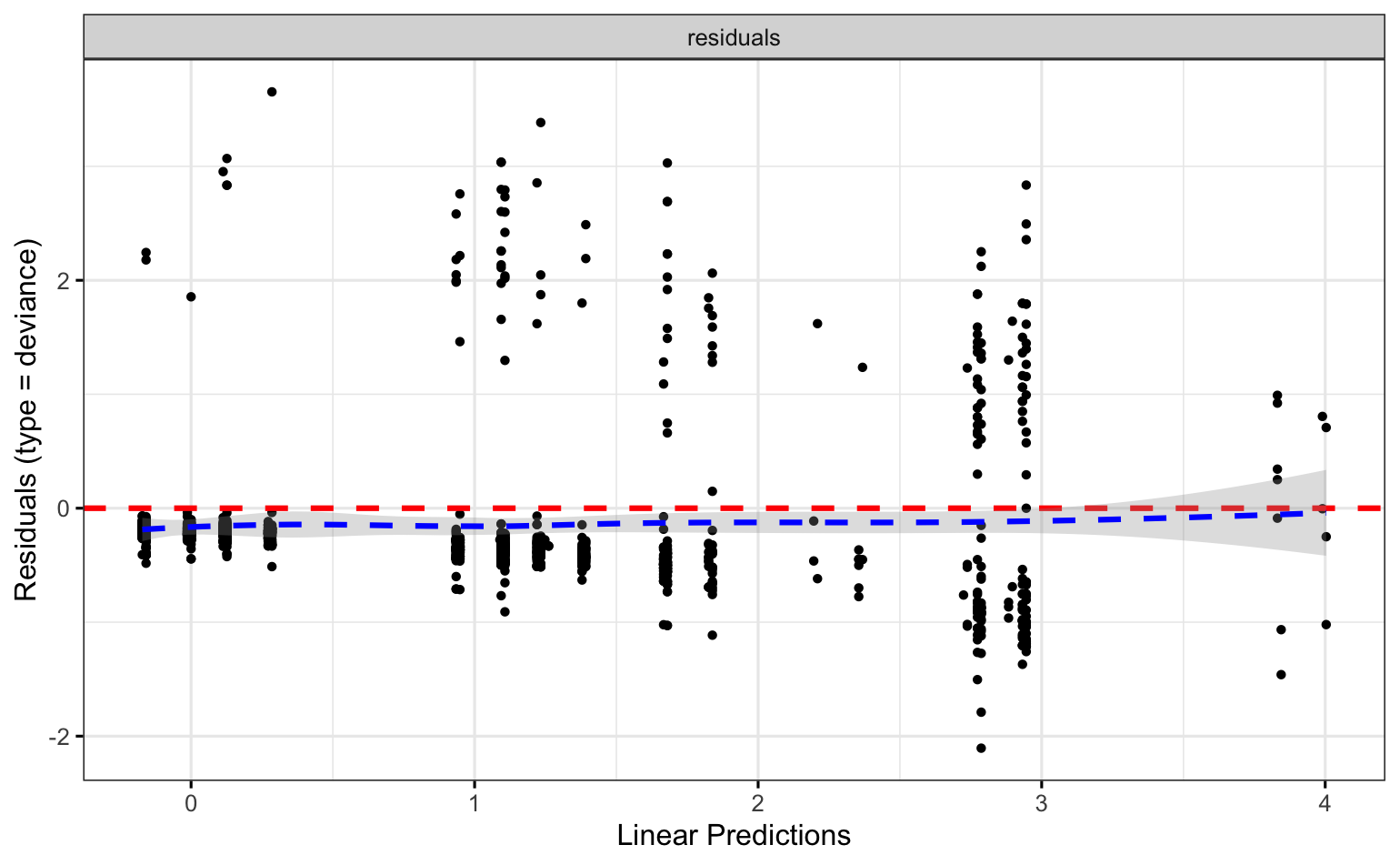

Supplement: Supplementary file 1 [file Table_1.DOCX]
